# Identification of Candidate lncRNA and Pseudogene Biomarkers Associated with Carbon-Nanotube-Induced Malignant Transformation of Lung Cells and Prediction of Potential Preventive Drugs

**DOI:** 10.3390/ijerph19052936

**Published:** 2022-03-02

**Authors:** Guangtao Chang, Dongli Xie, Jianchen Hu, Tong Wu, Kangli Cao, Xiaogang Luo

**Affiliations:** 1 College of Textile and Clothing Engineering, Soochow University, Suzhou 215123, China; changgt@suda.edu.cn (G.C.); xdl202111@163.com (D.X.); hujianchen@suda.edu.cn (J.H.); 2 Shanghai Jing Rui Yang Industrial Co., Ltd., Shanghai 200122, China; wutong@lead-all.cn; 3 Shanghai Institute of Spacecraft Equipment, Shanghai 200240, China; connieckl@126.com

**Keywords:** carbon nanotubes, malignant transformation, lung cancer, non-coding RNAs

## Abstract

Mounting evidence has linked carbon nanotube (CNT) exposure with malignant transformation of lungs. Long non-coding RNAs (lncRNAs) and pseudogenes are important regulators to mediate the pathogenesis of diseases, representing potential biomarkers for surveillance of lung carcinogenesis in workers exposed to CNTs and possible targets to develop preventive strategies. The aim of this study was to screen crucial lncRNAs and pseudogenes and predict preventive drugs. GSE41178 (small airway epithelial cells exposed to single- or multi-walled CNTs or dispersant control) and GSE56104 (lung epithelial cells exposed to single-walled CNTs or dispersant control) datasets were downloaded from the Gene Expression Omnibus database. Weighted correlation network analysis was performed for these two datasets, and the turquoise module was preserved and associated with CNT-induced malignant phenotypes. In total, 24 lncRNAs and 112 pseudogenes in this module were identified as differentially expressed in CNT-exposed cells compared with controls. Four lncRNAs (MEG3, ARHGAP5-AS1, LINC00174 and PVT1) and five pseudogenes (MT1JP, MT1L, RPL23AP64, ZNF826P and TMEM198B) were predicted to function by competing endogenous RNA (MEG3/RPL23AP64-hsa-miR-942-5p-CPEB2/PHF21A/BAMBI; ZNF826P-hsa-miR-23a-3p-SYNGAP1, TMEM198B-hsa-miR-15b-5p-SYNGAP1/CLU; PVT1-hsa-miR-423-5p-PSME3) or co-expression (MEG3/MT1L/ZNF826P/MT1JP-ATM; ARHGAP5-AS1-TMED10, LINC00174-NEDD4L, ARHGAP5-AS1/PVT1-NIP7; MT1L/MT1JP-SYNGAP1; MT1L/MT1JP-CLU) mechanisms. The expression levels and prognosis of all genes in the above interaction pairs were validated using lung cancer patient samples. The receiver operating characteristic curve analysis showed the combination of four lncRNAs, five pseudogenes or lncRNAs + pseudogenes were all effective for predicting lung cancer (accuracy >0.8). The comparative toxicogenomics database suggested schizandrin A, folic acid, zinc or gamma-linolenic acid may be preventive drugs by reversing the expression levels of lncRNAs or pseudogenes. In conclusion, this study highlights lncRNAs and pseudogenes as candidate diagnostic biomarkers and drug targets for CNT-induced lung cancer.

## 1. Introduction

Carbon nanotubes (CNTs), which are structurally composed of nano-sized hollow molecular cylinders rolled up from single (single-walled CNTs, SWCNTs) or multiple layers (multi-walled CNTs, MWCNTs) of graphene sheets, have become widely utilized in industrial and biomedical fields due to their unique properties [1,2,3,4,5]. However, their wide range of applications and mass production may lead to a concern that these CNTs can potentially enter the human body (especially for occupational workers) and cause adverse health consequences. Therefore, toxicity issues regarding CNTs have recently been the subject of much attention.

Inhalation is the primary route of human exposure to environmental pollutants, and the lungs are the major target organ for airborne exposure. Thus, inhalation of CNTs may result in an elevated risk for lung diseases, such as fatal lung cancer [6]. This theory has been observed in several in vitro and in vivo studies. Wang et al. found significant increases in the proliferation, migration and invasion abilities of human lung epithelial BEAS-2B cells and small airway epithelial cells (SAECs) exposed to SWCNTs and MWCNTs for 24 weeks [7,8]. Luanpitpong et al. demonstrated that exposure to SWCNTs and MWCNTs induced the transformation of primary human lung fibroblasts into cancer-associated fibroblasts and promoted the growth of human lung carcinoma H460 cells in mice [9]. Suzui et al. administered MWCNTs to rats and found the incidence of lung tumors was 14/38 in the MWCNT group but 0/28 in the control group after a 109-week follow-up period [10]. A study by Saleh et al. showed that administration of MWCNTs into rat lungs significantly elevated the incidence of lung tumors (7/20 vs. 1/19) compared with the vehicle group [11]. These findings indicate the necessity of investigating the carcinogenic mechanisms of CNTs to develop surveillance and prevention strategies.

Recently, studies have assessed the molecular mechanisms of CNT-induced malignant transformation of lungs. Voronkova et al. identified that exposure of BEAS-2B cells to SWCNTs for six months induced cell transformation by increasing the expression level of SOX9 protein [12]. Polimeni et al. reported that incubation of BEAS-2B cells with MWCNTs for 96 h could induce cell epithelial–mesenchymal transition (EMT) by activation of the transforming growth factor (TGF)-β-mediated signaling pathway [13]. Wang et al. confirmed that SWCNT-induced upregulation of Slug contributed to EMT, migration, invasion and anchorage-independent growth of BEAS-2B cells and tumor formation, as well as metastasis in xenograft mouse models [14]. Furthermore, the microarray or sequencing method was used to explore the genome-wide expression changes in CNT-transformed BEAS-2B cells. Upregulation of MYC, PPARG [8], c-FLIP [15], caveolin-1 [16], Ras family genes [17], CXCL8 and ADCY1 [18] and downregulation of cyclin E [19], FLG, SCN9A and TNS4 [18] were identified. A 35-gene signature was screened from mice 56 days after exposure to MWCNTs, and this signature was proven to predict the development of lung cancer from controls at a high accuracy rate (>60%) [20]. These findings suggest that these genes may be underlying biomarkers for medical surveillance of the lung cancer risk in occupational CNT-exposed workers and potential targets for the development of preventive and therapeutic drugs. However, the mechanisms linking CNT exposure to lung cancer is not well understood. More importantly, the genome not only consists of coding messenger RNAs (mRNAs) but also includes non-coding RNAs (ncRNAs), such as microRNAs (miRNAs), long ncRNAs (lncRNAs) and pseudogenes [21]. LncRNAs and pseudogenes can act as upstream factors to modulate the expression levels of mRNAs by directly regulating their transcription (that is, co-expression mechanism) or by sponging miRNAs and then influencing miRNA-mediated gene silencing (that is, competing endogenous RNA (ceRNA) mechanism) [21], indicating lncRNAs and pseudogenes may also represent crucial biomarkers and targets for CNT-induced lung cancer, which had not been reported previously.

In this study, we aimed to screen hub lncRNAs and pseudogenes linked to CNT-induced malignant transformation of human lung epithelial cells based on construction of ceRNA and co-expression networks and validation of the expression levels and prognosis of network genes. The drugs that reversed the expression levels of these lncRNAs and pseudogenes were also predicted. Our findings may provide new insights into predicting and preventing lung diseases of occupational workers with CNT exposure.

## 2. Materials and Methods

### 2.1. Data Acquisition and Preprocessing

The mRNA expression profiles of lung cancer induced by bare, hydrophobic CNT particles that were not wrapped in biocompatible materials were retrieved from the National Center for Biotechnology Information (NCBI) Gene Expression Omnibus (GEO) database (http://www.ncbi.nlm.nih.gov/geo/, accessed on 5 September 2021) by searching with the following key words: [“carbon nanotubes” AND (“lung carcinogenesis” OR “lung transformation”)], after which only two microarray datasets (GSE41178 [8] and GSE56104 [15]) were obtained. In the GSE41178 dataset, which is based on the platform of NimbleGen Homo sapiens Expression Array [100718_HG18_opt_expr] (GPL16025), whole-genome expression profiling was conducted on human immortalized SAECs following chronic exposure to dispersed SWCNTs (CNI, Houston, TX, USA; dry mean length, 1–4 μm; dispersed mean length, 1.08 μm; dry mean width, 1–4 nm; dispersed mean width, 270 nm; surface area, 400–1040 m^2^/g; metal contamination, <1%), MWCNTs (MWNT-7, Mitsui & Company, Tokyo, Japan; dry mean length, 8.19 ± 1.7 μm; dispersed mean length, 5.1 μm; dry mean width, 81 ± 5 nm; dispersed mean width, 78 nm; surface area, 26 m^2^/g; metal contamination, 0.78% [8]; the number of walls, 20–50 walls [22]) and Survanta^®^ dispersant (control) for six months (24 passages). A sub-toxic concentration (0.02 μg/cm^2^, equivalent to 0.1 μg/mL) was used for SWCNT and MWCNT exposure, which had been demonstrated to be either co-localize in the cytoplasm of cells or puncture the cellular or nuclear membranes and lead to increases in soft agar colony formation, proliferation, invasion, migration and angiogenesis of SAECs (malignant transformation phenotypes) [8]. Each treatment possessed three biological replicates. In the GSE56104 dataset, human immortalized BEAS-2B cells were exposed to Survanta^®^ dispersant or SWCNTs (CNI, Houston, TX, USA) with the similar concentration and characteristics as the GSE41178 dataset for six months. The proliferative, invasive, migration and angiogenic activities were increased, whereas apoptosis was reduced in BEAS-2B cells treated with SWCNTs [7,15]. Then, three biological replicates were collected for each treatment to perform whole-genome expression profiling on the platform of NimbleGen Homo sapiens HG18 expression array [100718_HG18_opt_expr_HX12] SEQ_ID condensed version (GPL18451). 

The expression matrix and gene-symbol annotation files of GSE41178 and GSE56104 datasets were downloaded from the GEO database. The gene type (belonging to lncRNAs, mRNAs or pseudogenes) was annotated according to the file retrieved from the NCBI Entrez Gene ftp site (ftp://ftp.ncbi.nlm.nih.gov/gene/DATA/; accessed on 5 September 2021).

### 2.2. Weighted Correlation Network Analysis of lncRNAs and Pseudogenes 

The weighted correlation network analysis (WGCNA) package in R (v1.61; https://cran.r-project.org/web/packages/WGCNA/index.html; accessed on 7 September 2021) [23] was employed to screen crucial lncRNAs and pseudogenes associated with CNT-induced phenotypic traits. The data of all overlapped lncRNAs and pseudogenes between GSE41178 and GSE56104 datasets were used to build the co-expression network. GSE41178 was set as the training dataset, and GSE56104 was set as the testing dataset. The filtering principle of the optimal soft threshold power (β) was to make the network have the scale-free topology characteristics. The weighted adjacency matrix was transformed into a topological overlap matrix to construct a dendrogram. The dynamic tree cut method was applied to detect gene modules with a minModuleSize of 50 and a cut height of 0.995. The preservation of identified modules was analyzed using the modulePreservation function, with a Z summary score > 2–10 defined as moderately preserved. The module eigengenes (ME) were calculated to represent the overall expression level of each module. The correlation between ME and the disease status was investigated in each module. If the correlation coefficient, r, was >0.5 and the *p*-value was <0.05, modules were considered crucial. Furthermore, the gene significance (GS) was measured to represent the correlation between the expression levels of genes in each module and each trait; module membership (MM) was measured to correlate the ME with gene expression values to determine the importance of a gene in a given module. LncRNAs and pseudogenes with |GS| > 0.5 and |MM| > 0.5 in crucial modules were considered hub genes.

### 2.3. Differential Analysis of lncRNAs, Pseudogenes and mRNAs in GSE41178 Dataset

The LIMMA (linear models for microarray data) package (v3.34.7; https://bioconductor.org/packages/release/bioc/html/limma.html; accessed on 10 September 2021) [24] was used to identify differentially expressed lncRNAs (DE-lncRNAs), pseudogenes (DE-pseudogenes) and mRNAs (DE-mRNAs) between SWCNT- or MWCNT-exposed and dispersant control-treated SAECs. The adjusted *p*-value < 0.05 and |log_2_fold change (FC)| > 0.5 were set as the cut-off point. The heatmaps of DE-lncRNAs, DE-pseudogenes and DE-mRNAs were plotted with R package “pheatmap” (v1.0.8; https://cran.r-project.org/web/packages/pheatmap; accessed on 10 September 2021). A Venn diagram (http://bioinformatics.psb.ugent.be/webtools/Venn/; accessed on 10 September 2021) was used to obtain the overlapped DE-lncRNAs, DE-pseudogenes and DE-mRNAs induced by SWCNTs and MWCNTs, as well as the overlapped DE-lncRNAs and DE-pseudogenes with hub genes in crucial modules identified by WGCNA analysis.

### 2.4. Validation of the Expression Levels and Prognosis of DE-lncRNAs and DE-Pseudogenes in Lung Cancer

To evaluate whether these hub DE-lncRNAs and DE-pseudogenes were associated with initiation of lung cancer, we validated their expression levels using Gene Expression Profiling Interactive Analysis (GEPIA, http://gepia.cancer-pku.cn; accessed on 12 September 2021), a newly developed interactive web server for analyzing the RNA sequencing expression data of 9736 tumors and 8587 normal samples from the Cancer Genome Atlas (TCGA) and the Genotype-Tissue Expression (GTEx) project [25]. Lung adenocarcinoma (LUAD) and squamous cell carcinoma (LUSC) samples were selected as the disease group; whereas match TCGA normal and GTEx data or match TCGA normal data were selected as the control group. |log_2_FC| > 0.5 and *p*-value < 0.05 were set as the cut-off point. 

To evaluate whether these hub DE-lncRNAs and DE-pseudogenes were associated with prognostic outcomes of lung cancer patients, the Kaplan–Meier plotting (http://www.kmplot.com/analysis/index.php?p=background; accessed on 12 September 2021) [26] was performed. Kaplan–Meier plots allow for assessment of the effects of DE-lncRNAs and DE-pseudogenes (included in mRNA data) on survival using the GeneChip and RNA-seq data of GEO, the European Genome-Phenome Archive and TCGA databases. The overall survival (OS) and recurrence-free survival (RFS) was assessed by RNA-seq data analysis, whereas OS, first-progression survival (FPS) and post-progression survival (PPS) were assessed in GeneChip data analysis. Patients were divided into two groups by “auto-select best cut-off”. Hazard ratios (HRs) with 95% confidence intervals (CIs) and log-rank *p*-values were automatically calculated. A log-rank test *p*-value < 0.05 was considered statistically significant. HR < 1 indicated a better survival rate for the high-expression group; HR > 1 indicated a poor survival rate for the high-expression group.

### 2.5. Construction of lncRNA- and Pseudogene-Related ceRNA Networks

The DE-lncRNAs and DE-pseudogenes with validated expression and survival associations in lung cancer data were used to construct the ceRNA networks. The interactions between DE-lncRNAs/DE-pseudogenes and miRNAs were predicted using the starBase database (v2.0; http://starbase.sysu.edu.cn/; accessed on 26 September 2021) [27] with the cut-off point of Ago CLIP-seq Data ≥ 3. Then, miRNAs that interacted with lncRNAs/pseudogenes were used to predict their target mRNAs by the starBase database, with the cut-off point of predicting program ≥ 3 and CLIP data > 3. The final lncRNA/pseudogene–miRNA–mRNA interaction pairs were confirmed after comparing the predicted mRNAs and DE-mRNAs. Only DE-mRNAs that had expression trends similar to those of lncRNAs or pseudogenes were retained. The ceRNA network was visualized in Cytoscape (v3.6.1; www.cytoscape.org/; accessed on 26 September 2021). 

### 2.6. Construction of lncRNA- and Pseudogene-mRNA Co-Expression Networks

The DE-lncRNAs and DE-pseudogenes with validated expression and survival associations in lung cancer data were used to construct the co-expression networks. The Pearson correlation coefficient (PCC) was calculated between DE-lncRNAs/DE-pseudogenes and DE-mRNAs. Meaningful correlation pairs were defined as PCC > 0.9 and *p*-value < 0.05. The co-expression network was visualized using Cytoscape.

### 2.7. Construction of Protein–Protein Interaction (PPI) Networks

To further screen hub DE-mRNAs from ceRNA and co-expression networks, we respectively imported DE-mRNAs of lncRNA- and pseudogene-related networks into the Search Tool for the Retrieval of Interacting Genes (STRING; v10.0; http://stringdb.org/; accessed on 26 September 2021) database [28] to obtain the PPI pairs. PPI pairs with a combined score > 0.4 were considered significant. Furthermore, seven topological characteristics of each protein in the PPI network were calculated using the CytoNCA plugin in Cytoscape software (http://apps.cytoscape.org/apps/cytonca; accessed on 26 September 2021) [29], including degree, betweenness, closeness, eigenvector, local average connectivity, information and subgragh centrality. The proteins ranked in the top 60 of each topological characteristic were suggested to be hub DE-mRNAs.

### 2.8. Validation of the Expression Levels and Survival Associations of DE-mRNAs in Lung Cancer

The mRNA expression levels of hub DE-mRNAs were confirmed using the GEPIA database as lncRNAs and pseudogenes. The protein expression levels of hub DE-mRNAs were validated using UALCAN (http://ualcan.path.uab.edu/index.html; accessed on 26 September 2021) (in which proteomic datasets of LUAD and normal controls from the Clinical Proteomic Tumor Analysis Consortium (CPTAC) were provided; *p*-value < 0.05 was set as the significant threshold value) and the Human Protein Atlas (HPA; v20.1, https://www.proteinatlas.org; accessed on 27 September 2021) (in which immunohistochemistry results for LUAD and LUSC tissues were available). The associations of hub DE-mRNAs with OS, RFS, FPS and PPS were analyzed using the Kaplan–Meier plotter based on the sequencing and chip data.

### 2.9. Validation of the Expression Levels and Survival Associations of miRNAs in Lung Cancer

The expression levels of miRNAs associated with hub DE-lncRNAs and DE-mRNAs were confirmed using the UALCAN database (http://ualcan.path.uab.edu/index.html; accessed on 6 October 2021) based on the TCGA data of LUAD and LUSC patient samples. Furthermore, Ventura et al. performed an miRNA expression profile analysis of A549 cells exposed to MWCNTs (MWCNT-7, Mitsui & Company, Ibaraki, Japan) and controls [30]. The differentially expressed miRNAs identified in the study of Ventura et al. were also compared with ours to confirm the expression levels of crucial miRNAs induced by CNTs. The associations of miRNAs with OS and RFS were identified using the Kaplan–Meier plotter based on the sequencing data.

### 2.10. Function Enrichment Analysis

To understand potential functions of mRNAs regulated by DE-lncRNAs and DE-pseudogenes, the online Database for Annotation, Visualization and Integrated Discovery (DAVID) (v6.8; http://david.abcc.ncifcrf.gov; accessed on 12 October 2021) [31] was searched, after which gene ontology (GO) biological process, molecular function terms and Kyoto Encyclopedia of Genes and Genomes (KEGG) pathways were obtained at a significance level of *p*-value < 0.05. The “Goplot” package in R was used to draw the chord plot for hub DE-mRNAs.

### 2.11. Diagnostic Analysis of Hub lncRNAs and Pseudogenes

The mRNA sequencing data of LUAD/LUSC patients and normal controls were downloaded from the TCGA database. The receiver operating characteristic (ROC) curve analysis was performed using the MedCalc program (v9.3; MedCalc, Mariakerke, Belgium) to assess the diagnostic value of hub lncRNAs and pseudogenes in distinguishing lung cancer from normal controls. The area under the ROC curve (AUC), sensitivity and specificity were calculated. The genes with AUC > 0.5 were considered to have diagnostic significance.

### 2.12. Prediction of Small-Molecule Drugs That Regulated Hub lncRNAs and Pseudogenes

To predict potential drugs that may prevent and treat CNT-induced lung cancer, the Comparative Toxicogenomics Database (CTD, http://ctdbase.org; accessed on 12 October 2021) [32] was searched to obtain the chemical interactions of hub lncRNAs and pseudogenes associated with CNT exposure. Only chemical drugs that reversed the expression levels of lncRNAs and pseudogenes and involved in cancer initiation and development were screened (that is, if the lncRNA was upregulated, we selected the drugs that decreased its expression levels). The lncRNA/pseudogene–chemical interaction network was visualized in Cytoscape.

## 3. Results

### 3.1. Identification of Key Modules Related to CNT-Induced Malignant Transformation of Lung Cells

After Entrez Gene annotation, the GSE41178 dataset was found to include 995 lncRNAs (726) and pseudogenes (269), whereas the GSE56104 dataset included 1051 lncRNAs (305) and pseudogenes (746). A total of 812 lncRNAs and pseudogenes were shared between the GSE41178 and GSE56104 datasets. Additionally, the expression levels (r = 0.87, *p* < 1 × 10^200^) and connectivity (r = 0.13, *p* = 2 × 10^4^ were significantly positively correlated between these two datasets. Thus, these shared 812 lncRNAs and pseudogenes were used for WGCNA analysis.

**Figure 1:**
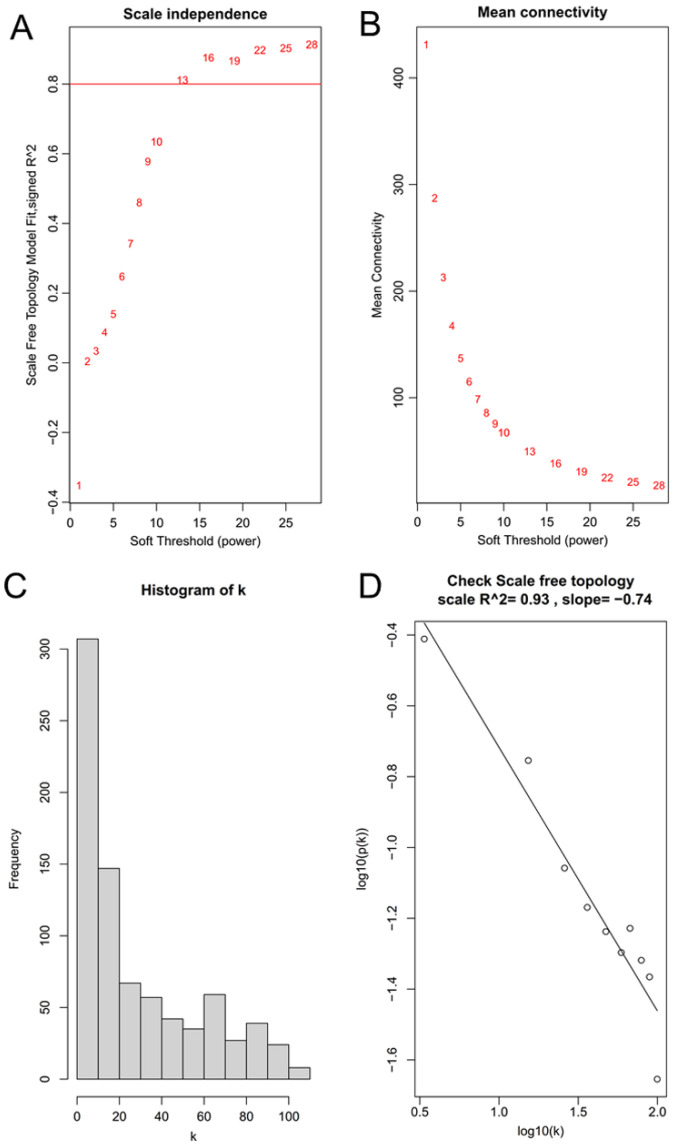
Determination of the best soft-threshold power in the WGCNA. (**A**) Analysis of the scale-free fit index for various soft-threshold powers (from 1 to 28); (**B**) analysis of the mean connectivity for various soft-threshold powers (from 1 to 28); (**C**) the histogram of k; (**D**), the correlation coefficient between k and *p* (k) to check the scale-free topology when β = 13.

**Figure 2:**
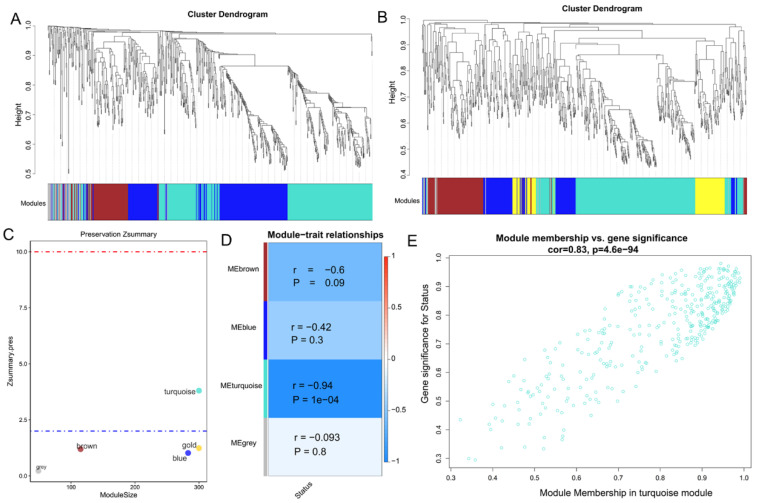
Identification of modules associated with CNT-induced malignant transformation of lung cells. (**A**) A clustering dendrogram obtained from data of the GSE41178 dataset; (**B**) a clustering dendrogram obtained from data of the GSE56104 dataset; (**C**) the preservation median rank of co-expression modules; (**D**): module–trait associations. Each row corresponds to a module eigengene (ME) and each column to a trait. Each cell contains the corresponding correlation (r) and *p*-value; (**E**) scatter plot showing the correlation of gene significance versus module membership for genes in the turquoise module.

A β value of 13 was selected to satisfy the requirements of the scale-free network distribution (Figure 1A,B). By calculating the scale-free topology fitting index, the value of R square was confirmed to reach 0.93 (Figure 1C,D). After a dynamic tree cut analysis, four modules were discovered using the GSE41178 training dataset (Figure 2A). These modules were also generated after analysis with the GSE56104 testing dataset (Figure 2B). Among them, only the turquoise module was moderately preserved (Z summary score = 4.21; Figure 2C). The module–trait heatmap revealed that the eigengenes of the turquoise module were strongly correlated with disease status (r = −0.94, *p* = 1 × 10^8^; Figure 2D). The correlation between GS for disease status and MM in the turquoise module was also significant (r = 0.83, *p* = 4.61 × 10^94^; Figure 2E). These findings indicated that 66 lncRNAs and 299 pseudogenes in this module were highly correlated with CNT-induced malignant transformation of lung cells. Among them, 57 lncRNAs and 267 pseudogenes had |GS| > 0.5 and |MM| > 0.5. Thus, they were considered to be hub lncRNAs and pseudogenes and chosen for further analyses.

### 3.2. Identification of Differential Genes in CNT-Induced Malignant Transformation of Lung Cells

**Figure 3:**
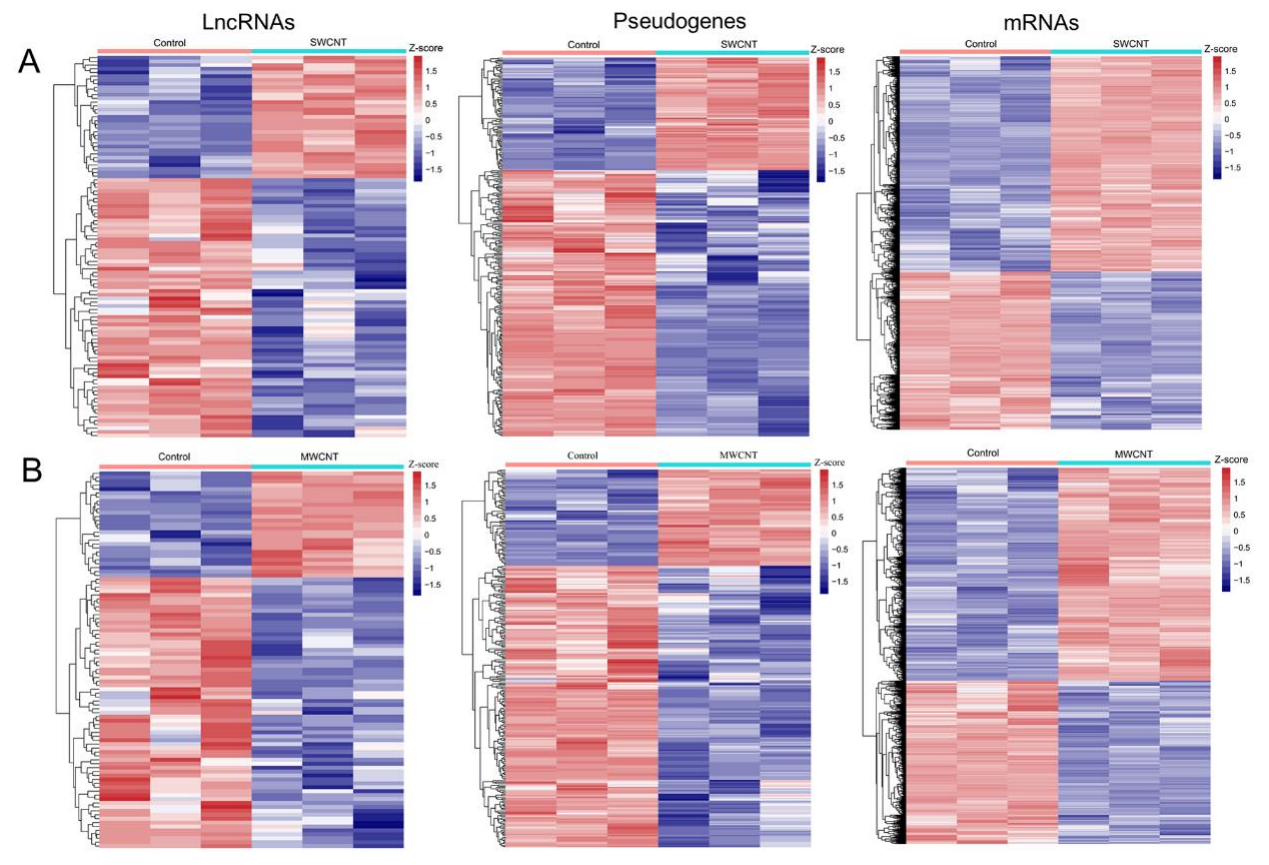
Heatmap of the expression levels of all differentially expressed lncRNAs, pseudogenes and mRNAs between CNT-exposed and control SAECs. (**A**) The difference between SWCNT-exposed and control SAECs; (**B**) the difference between MWCNT-exposed and control SAECs. SWCNT, single-walled carbon nanotube; MWCNT, multiple-walled carbon nanotube; red, high expression; blue, low expression.

Using theLIMMA method, a total of 103 lncRNAs (33 upregulated, 70 downregulated), 289 pseudogenes (86 upregulated, 203 downregulated) and 5136 mRNAs (2961 upregulated, 2175 downregulated) were identified to be differentially expressed between SWCNT-exposed and control-treated SAECs. A total of 99 lncRNAs (27 upregulated, 72 downregulated), 276 pseudogenes (70 upregulated, 206 downregulated) and 4695 mRNAs (2659 upregulated, 2036 downregulated) were identified to be differentially expressed between MWCNT-exposed and control-treated SAECs. The heatmaps of DE-lncRNAs, DE-pseudogenes and DE-mRNAs are displayed in Figure 3. A Venn diagram showed that there were 63 DE-lncRNAs (15 upregulated, 48 downregulated), 203 DE-pseudogenes (47 upregulated, 156 downregulated) and 3348 DE-mRNAs (1976 upregulated, 1372 downregulated) shared between SWCNTs and MWCNTs (Figure S1A,B). Furthermore, we also compared these DE-lncRNAs and DE-pseudogenes with hub lncRNAs and pseudogenes identified in WGCNA analysis. The results showed that 24 lncRNAs and 112 pseudogenes were overlapped (Figure S1C). These findings suggest that these 24 DE-lncRNAs and 112 DE-pseudogenes may be particularly important lncRNAs and pseudogenes associated with CNT-induced malignant transformation of lung cells.

### 3.3. Validation of the Expression Levels and Prognosis of lncRNAs and Pseudogenes in Tissues of Lung Cancer Patients

**Figure 4:**
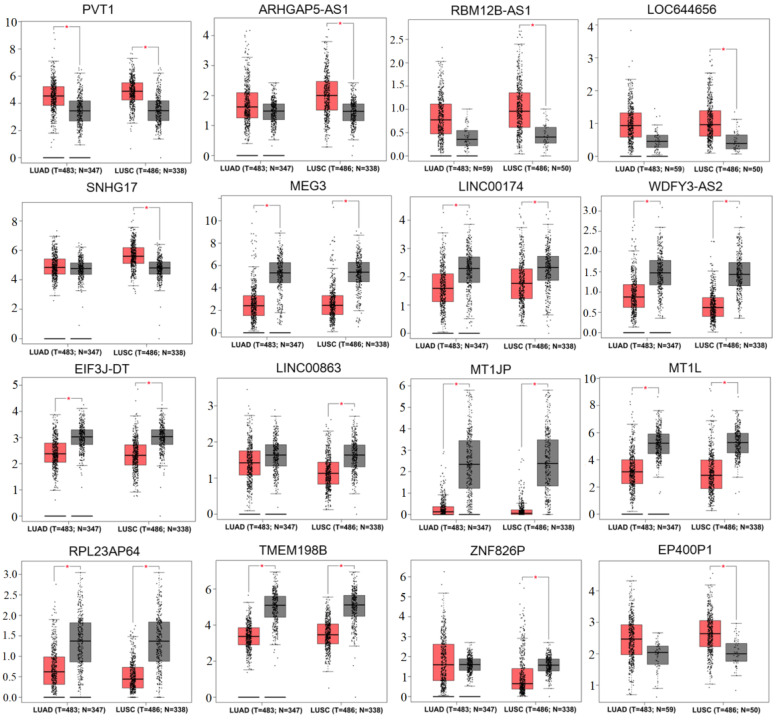
Validation of the expression levels of lncRNAs and pseudogenes in lung cancer. N = 50 or 59 indicates the controls are match TCGA normal data; N = 338 or 347 indicates the controls are match TCGA normal and GTEx data; asterisk (*) indicates the statistical significance at the threshold of |log_2_FC| > 0.5 and *p*-value < 0.05.

**Figure 5:**
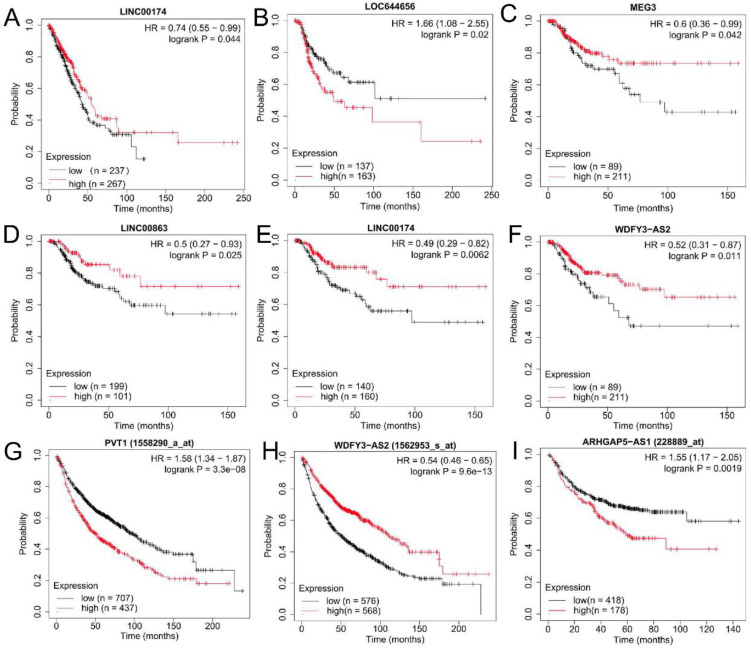
Validation of the prognosis of lncRNAs in lung cancer. (**A**) LINC00174. Prediction for overall survival of LUAD patients by analysis of TCGA data; (**B**) LOC644656. Prediction for recurrence-free survival of LUAD patients by analysis of TCGA data; (**C**–**F**) MEG3 (**C**), LINC00863 (**D**), LINC00174 (**E**) and WDFY3-AS2 (**F**). Prediction for recurrence-free survival of LUSC patients by analysis of TCGA data; (**G**,**H**) PVT1 (**G**) and WDFY3-AS2 (**H**). Prediction for overall survival of all lung cancer patients by analysis of chip data; (**I**) ARHGAP5-AS1. Prediction for first-progression survival of all lung cancer patients by analysis of chip data. HR, hazard ratio; LUAD, lung adenocarcinoma; LUSC, lung squamous cell carcinoma; TCGA, the Cancer Genome Atlas.

**Figure 6:**
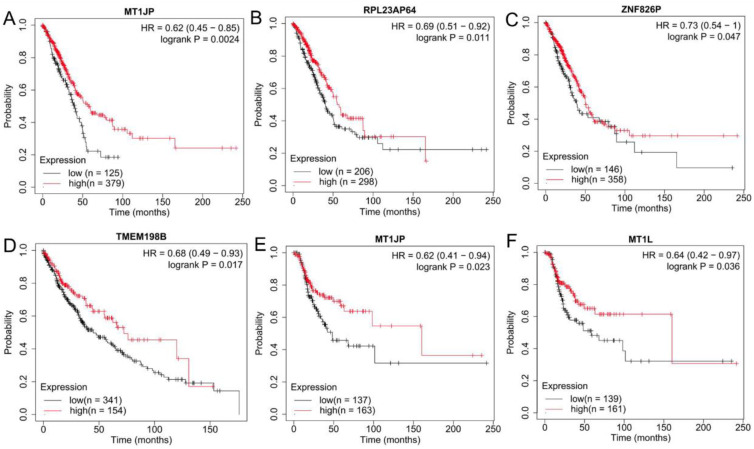
Validation of the prognosis of pseudogenes in lung cancer. (**A**–**C**) MT1JP (**A**), RPL23AP64 (**B**) and ZNF826P (**C**). Prediction for overall survival of LUAD patients by analysis of TCGA data; (**D**) TMEM198B. Prediction for overall survival of LUAD patients by analysis of TCGA data; (**E**,**F**) MT1JP (**E**) and MT1L (**F**). Prediction for recurrence-free survival of LUSC patients by analysis of TCGA data. HR, hazard ratio; LUAD, lung adenocarcinoma; LUSC, lung squamous cell carcinoma; TCGA, the Cancer Genome Atlas.

Under the threshold of log_2_FC > 0.5 and *p*-value < 0.5, the GEPIA database confirmed that PVT1 was significantly upregulated in both LUAD and LUSC samples compared with controls; ARHGAP5-AS1, RBM12B-AS1, LOC644656 and SNHG17 were only more highly expressed in LUSC samples compared to controls; MEG3, LINC00174, WDFY3-AS2 and EIF3J-DT were significantly downregulated in both LUAD and LUSC samples compared with controls; LINC00863 was only had lower expression in LUSC samples compared to controls (Figure 4). The expression trend was in line with the results of these lncRNAs identified in the GSE41178 dataset (Table 1). Kaplan–Meier plotter analysis demonstrated that patients with high expression levels of PVT1, ARHGAP5-AS1 and LOC644656 possessed a shorter survival rate (HR >1), whereas high expression levels of MEG3, LINC00174, WDFY3-AS2 and LINC00863 were associated with excellent prognostic outcomes (HR< 1) (Figure 5; Table S1). The prognostic results of these lncRNAs were in accordance with our expectation according to their expression trends. Likewise, GEPIA and the Kaplan–Meier plotter database were used to confirm the expression levels and prognosis of pseudogenes, respectively. As a result, MT1JP, MT1L, RPL23AP64 and TMEM198B were found to be significantly downregulated in both LUAD and LUSC samples compared with controls; ZNF826P was downregulated and EP400P1 was upregulated in LUSC samples compared with controls (Figure 4), which was in line with the results obtained in the GSE41178 dataset (Table 1). Among these six pseudogenes, five (HR <1 in all pseudogenes) were significantly associated with prognostic outcomes of lung cancer patients (Figure 6). These findings suggest that these seven lncRNAs and five pseudogenes may be potentially important genes associated with CNT-induced initiation and progression of lung cancer, which were used for the following ceRNA and co-expression network analyses.

### 3.4. Establishment of lncRNA- and Pseudogene-Based ceRNA Regulatory Networks

The starBase database predicted that five lncRNAs (LINC00174, LINC00863, MEG3, ARHGAP5-AS1 and PVT1) could interact with 41 miRNAs, and among these 41 miRNAs, 32 could target 3429 mRNAs. Integration of these lncRNA–mRNA and miRNA–mRNA interaction pairs and selection of the intersection of predicted mRNAs with DE-mRNAs showed that upregulated PVT1 could interact with 15 miRNAs to lead to the upregulation of 376 DE-mRNAs, whereas downregulated LINC00174, LINC00863 and MEG3 could interact with 15 miRNAs to result in the downregulation of 88 DE-mRNAs. Thus, these four DE-lncRNAs-30, miRNAs-464 and DE-mRNAs were used to establish an lncRNA-related ceRNA network (Table S2).

The starBase database predicted that three downregulated pseudogenes (RPL23AP64, ZNF826P and TMEM198B) could interact with 36 miRNAs, whereas 35 miRNAs could target 3053 mRNAs (among which 146 were downregulated). Thus, three DE-pseudogenes-27, miRNAs-146 and DE-mRNAs were ultimately used to establish a pseudogene-related ceRNA regulatory network (Table S2).

### 3.5. Establishment of lncRNA- and Pseudogene-Based Co-Expression Networks

Under the threshold of PCC > 0.9 and *p*-value < 0.05, a total of 402 co-expression pairs were obtained, including the interactive relationships between three upregulated DE-lncRNAs (PVT, ARHGAP5-AS1 and LOC64465) and 161 upregulated DE-mRNAs, as well as between four downregulated DE-lncRNAs (MEG3, WDFY3-AS2, LINC00863 and LINC00174) and 192 downregulated DE-mRNAs (Table S4); for pseudogenes, a total of 455 co-expression pairs, which were constructed by five downregulated DE-pseudogenes (MT1JP, MT1L, TMEM198B, ZNF826P and RPL23AP64) and 302 downregulated DE-mRNAs, were identified (Table S3).

### 3.6. Establishment of PPI Networks for lncRNA- and Pseudogene-Related Target Genes

To screen crucial target mRNAs regulated by lncRNAs and pseudogenes, we attempted to explore the interactions of mRNAs in the ceRNA and co-expression networks by using the STRING database. As a result, 2527 PPI pairs with a combined score > 0.4, were obtained for target mRNAs of lncRNAs, which were constructed by 685 genes, whereas 286 genes and 513 PPI pairs were predicted for target mRNAs of pseudogenes (Table S4). The hub genes were selected according to the top 60 genes ranked in seven topological characteristics, which led to 112 genes screened from lncRNA- (Table 2) and pseudogene-related (Table 3) PPI pairs, respectively.

### 3.7. Validation of the Expression Levels and Prognosis of lncRNA- and Pseudogene-Related mRNAs and miRNAs in Lung Cancer

To screen crucial interaction axes, we compared the miRNAs and mRNAs in the ceRNA network, co-expression network and the top 60 genes of the PPI network. The results showed that hsa-miR-942-5p was the only overlapped miRNA included in the ceRNA networks of lncRNAs and pseudogenes. Thus, MEG3/RPL23AP64-hsa-miR-942-5p-cytoplasmic polyadenylation element binding protein 2 (CPEB2)/PHD finger protein 21A (PHF21A)/BMP and activin membrane-bound inhibitor (BAMBI) interactive axes were potentially important; ATM serine/threonine kinase (ATM) was the target gene that could be regulated by both of lncRNA MEG3 and pseudogene MT1L/ZNF826P in the co-expression network and was ranked as the hub gene in the PPI networks of both lncRNAs and pseudogenes. The intersection analysis of mRNAs in the ceRNA, co-expression and PPI networks of lncRNAs identified three mRNAs (transmembrane p24 trafficking protein 10, TMED10; NEDD4 like E3 ubiquitin protein ligase, NEDD4L; and nucleolar pre-rRNA processing protein NIP7, NIP7), and their corresponding relationship pairs were ARHGAP5-AS1-TMED10, PVT1-hsa-miR-214-3p/hsa-miR-873-5p-TMED10, LINC00174-NEDD4L, LINC00174-hsa-miR-519a-3p/hsa-miR-519b-3p/hsa-miR-519c-3p-NEDD4L, PVT1-hsa-miR-873-5p-NIP7 and ARHGAP5-AS1/PVT1-NIP7; the intersection analysis of mRNAs in the ceRNA, co-expression and PPI networks of pseudogenes identified three mRNAs (synaptic Ras GTPase activating protein 1, SYNGAP1; clusterin, CLU; and serum/glucocorticoid regulated kinase family member 3, SGK3), and their corresponding relationship pairs were MT1L-SYNGAP1, TMEM198B-hsa-miR-15a-5p/hsa-miR-15b-5p/hsa-miR-16-5p/hsa-miR-195-5p/hsa-miR-424-5p/hsa-miR-497-5p-SYNGAP1, ZNF826P-hsa-miR-23a-3p/hsa-miR-23b-3p-SYNGAP1, TMEM198B-hsa-miR-15a-5p/hsa-miR-15b-5p/hsa-miR-16-5p/hsa-miR-195-5p/hsa-miR-424-5p/hsa-miR-497-5p-CLU, MT1L-CLU, RPL23AP64-hsa-miR-376a-3p/hsa-miR-376b-3p-SGK3 and MT1JP/MT1L/RPL23AP64/ZNF826P-SGK3.

GEPIA analysis confirmed that the mRNA expression levels of CPEB2, PHF21A, BAMBI, ATM, NEDD4L, SYNGAP1, CLU and SGK3 were significantly lower, whereas TMED10 and NIP7 were significantly higher in both LUAD and LUSC samples than those in the normal controls (Figure S2). UALCAN analysis with the proteomic data validated that the protein expression levels of CPEB2, NEDD4L, SYNGAP1, CLU and SGK3 were significantly downregulated, whereas TMED10 and NIP7 were significantly upregulated in LUAD samples compared with controls (Figure S3). HPA immunohistochemical results revealed that PHF21A was expressed at medium levels in normal tissues but at medium to not-detected levels in LUAD and LUSC tissues. BAMBI was expressed at low levels in normal tissues but at not-detected levels in most LUAD and LUSC tissues. ATM was expressed at high levels in normal tissues but at medium-low levels in LUAD and medium to not-detected levels in most LUSC tissues. TMED10 was expressed at low levels in normal tissues but at high-medium levels in all LUAD samples and at medium levels in most LUSC tissues. SGK3 was expressed at low levels in normal tissues but at not-detected levels in LUAD and LUSC tissues (Figure S4). Kaplan–Meier plotter analysis demonstrated that there were significant associations between these 10 mRNAs and prognostic outcomes (Figure S5 and Table S5).

For miRNAs on interactive axes, only hsa-miR-942, hsa-miR-23a and has-miR-15b were validated to be differentially expressed and associated with prognosis after UALCAN and Kaplan–Meier plotter analyses (Figure S6). Consistent with TCGA analysis results, hsa-miR-23a-3p (log_2_FC = 0.69, *p* = 2.89 × 10^3^) and has-miR-15b-5p (log_2_FC = 0.62, *p* = 2.71 × 10^4^) were also detected to be upregulated in A549 cells after 24 h of exposure to MWCNTs [30]. Although the expression level of hsa-miR-423 (non-significant for LUAD; upregulated in LUSC) in TCGA data were not in line with our expectations, hsa-miR-423-5p was identified to be downregulated in A549 cells after 24 h of exposure to MWCNTs (log_2_FC = −0.76, *p* = 3.10 × 10^16^) (Figure S7). Additionally, Kaplan–Meier plotter analysis proved that high levels of hsa-miR-423 contributed to a longer survival rate (Figure S7). Thus, we considered that hsa-miR-423-5p-related ceRNA axes may also be pivotal. As the target mRNA of hsa-miR-423-5p, PSME3 was included in hub genes of the PPI network. PSME3 had significantly higher expression in LUAD and LUSC samples and predicted a poor prognosis (Figure S7).

**Figure 7:**
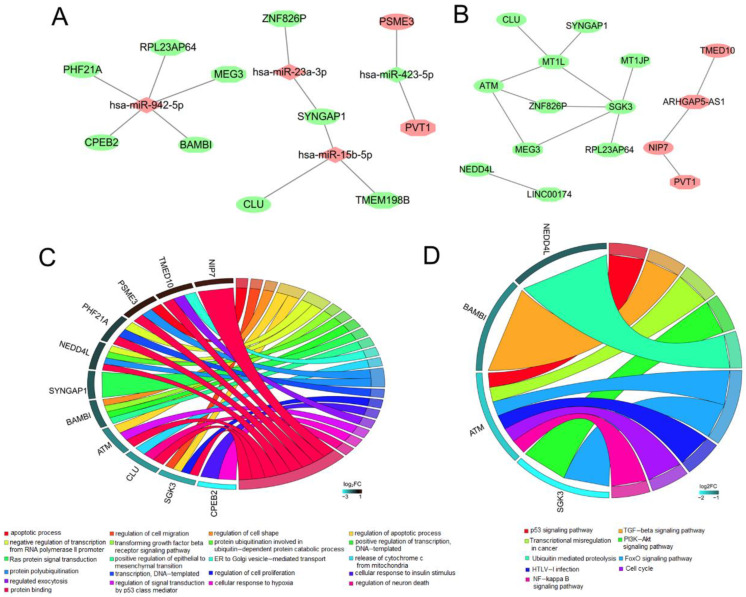
Functions of hub lncRNAs and pseudogenes. (**A**) ceRNA regulatory networks for hub lncRNAs and pseudogenes; (**B**) co-expression regulatory networks for hub lncRNAs and pseudogenes; (**C**) Gene ontology for target mRNAs of hub lncRNAs and pseudogenes; (**D**) KEGG pathway enrichment analysis for target mRNAs of hub lncRNAs and pseudogenes. ceRNA, competing endogenous RNA. Hexagon indicates lncRNAs and pseudogenes; rhombus indicates miRNAs; ellipse indicates mRNAs; red, upregulation; green, downregulation.

In summary, we believe that lncRNAs MEG3, ARHGAP5-AS1, LINC00174, PVT1 and pseudogenes MT1JP, MT1L, RPL23AP64, ZNF826P and TMEM198B may be crucial for CNT-induced lung carcinogenesis by the following interaction mechanisms: MEG3/RPL23AP64-hsa-miR-942-5p-CPEB2/PHF21A/BAMBI; ZNF826P-hsa-miR-23a-3p-SYNGAP1, TMEM198B-hsa-miR-15b-5p-SYNGAP1/CLU; PVT1-hsa-miR-423-5p-PSME3 (Figure 7A); MEG3/MT1L/ZNF826P-ATM; ARHGAP5-AS1-TMED10, LINC00174-NEDD4L, ARHGAP5-AS1/PVT1-NIP7; MT1L-SYNGAP1; MT1L-CLU; MEG3/MT1JP/MT1L/RPL23AP64/ZNF826P-SGK3 (Figure 7B).

### 3.8. Function Enrichment Analysis

All genes in PPI networks of lncRNAs and pseudogenes were uploaded into the DAVID database to predict their potential functions. A total of 125 GO biological process, 43 molecular function terms and 23 KEGG pathways were enriched (Table S6). The mRNAs regulated by crucial lncRNAs and pseudogenes were predicted to be involved in GO:0006915~apoptotic process (PSME3), GO:0030334~regulation of cell migration (SGK3), GO:0007179~transforming growth factor beta receptor signaling pathway, GO:0010718~positive regulation of epithelial to mesenchymal transition (BAMBI), GO:0007265~Ras protein signal transduction (SYNGAP1), GO:0006888~ER to Golgi vesicle-mediated transport (TMED10), GO:0000209~protein polyubiquitination (NEDD4L), GO:0006351~transcription, DNA-templated (PHF21A), GO:0071456~cellular response to hypoxia (CPEB2), GO:0001836~release of cytochrome c from mitochondria (CLU), O:0005515~protein binding (NIP7) (Figure 7C), hsa04115:p53 signaling pathway (ATM), hsa04350:TGF-beta signaling pathway (BAMBI), hsa04068:FoxO signaling pathway (ATM, SGK3), hsa04064:NF-kappa B signaling pathway (ATM), etc. (Figure 7D).

### 3.9. Diagnostic Values of Hub lncRNAs and Pseudogenes

**Figure 8:**
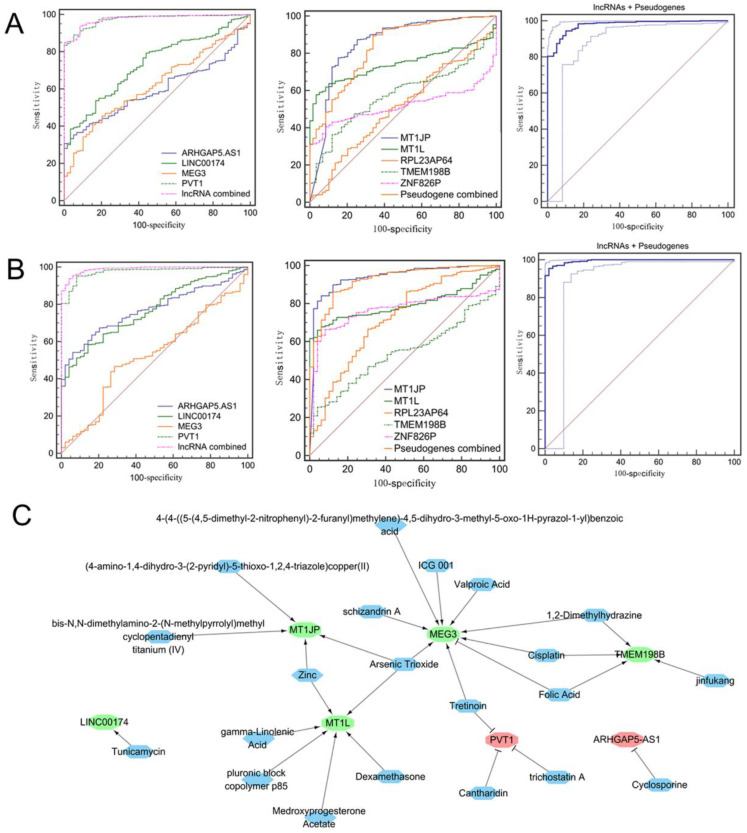
Diagnostic biomarkers and drug targets. (**A**,**B**) Diagnostic potential analysis of hub lncRNAs and pseudogenes. (**A**) Analysis of LUAD samples in TCGA database; (**B**) analysis of LUSC samples in TCGA database; (**C**) Drugs that reversed the expression levels of hub lncRNAs and pseudogenes in cancer, increased the expression or decreased the expression. Red, upregulated lncRNAs and pseudogenes; green, downregulated lncRNAs and pseudogenes; blue, drugs. LUAD, lung adenocarcinoma; LUSC, lung squamous cell carcinoma; TCGA, the Cancer Genome Atlas.

ROC analysis was performed on these four lncRNAs (MEG3, ARHGAP5-AS1, LINC00174 and PVT1) and five pseudogenes (MT1JP, MT1L, RPL23AP64, ZNF826P and TMEM198B) to explore their diagnostic values. The results showed that the AUCs of all single lncRNAs and pseudogenes were >0.5, indicating they had diagnostic significance in distinguishing lung cancer from normal controls. PVT1 and MT1JP may be especially effective because their AUCs were larger than 0.85 in the analysis of LUAD and LUSC samples (Figure 8A,B). Furthermore, the combination of all lncRNAs, pseudogenes and lncRNAs + pseudogenes was shown to further increase the AUC, sensitivity or specificity. Thus, this gene combination should be considered as a promising diagnostic biomarker (Figure 8A,B; Table 4).

### 3.10. Prediction of Small-Molecule Drugs That Regulated Hub lncRNAs and Pseudogenes

CTD analysis identified a series of drugs that reversed the expression levels of hub lncRNAs and pseudogenes in cancer (Figure 8C). Among them, schizandrin A (targeted MEG3), folic acid (targeted MEG3 and TMEM198B), zinc (targeted MT1JP and MT1L) and gamma-linolenic acid (targeted MT1L) may be candidate drugs to prevent the development of lung cancer for CNT occupational workers because they were components of traditional Chinese medicine or OTC health products.

## 4. Discussion

In recent decades, mounting evidence has supported the idea that environmental pollutants (such as PM2.5 [33,34,35], nickel [36] and cadmium [37,38]) promote the malignant transformation of lung epithelial cells by changing the expression levels of ncRNAs. Dysregulation of ncRNA expression levels was also revealed to be a novel paradigm for interpretation of the toxicology of various nanoparticles [39,40,41]. However, to date, there has been no study exploring the molecular mechanisms associated with CNT-induced lung carcinogenesis from the perspective of lncRNAs and pseudogenes. In this study, we, for the first time, attempted to screen potential lncRNAs and pseudogenes associated with CNT-induced lung carcinogenesis based on a comprehensive bioinformatics analysis of microarray data of lung cells directly exposed to CNTs without other influencing factors (GSE41178 and GSE561040) and sequencing (TCGA), proteomics (CPTAC) and immunohistochemical data (HPA) of lung cancer patients. Similar to the studies of PM2.5 [35], nickel [36] and cadmium [37] exposure, we also identified that the upregulation of PVT1 and downregulation of MEG3 contributed to the acquisition of tumorigenic phenotypes of human SAECs after CNT exposure. An increase in the expression level of lncRNA ARHGAP5-AS1 and decreases in the expression levels of lncRNA LINC00174, pseudogene MT1JP, MT1L, RPL23AP64, ZNF826P and TMEM198B were newly identified mechanisms in our study to explain the carcinogenic effects of CNTs. Nevertheless, some of our newly identified lncRNAs and pseudogenes had been demonstrated to mediate cancer development and progression. For example, Zhu et al. identified that ARHGAP5-AS1 was upregulated in chemo-resistant gastric cancer cells, and its knockdown reversed the chemo-resistance. A high expression level of ARHGAP5-AS1 was associated with a poor prognosis for gastric cancer patients [42]. The expression level of MT1JP was shown to be significantly lower in gastric cancer [43], hepatocellular carcinoma [44] and lung cancer [45] tissues (especially for lymph node metastasis and advanced-stage cancer) than that in adjacent normal tissues. The survival time was significantly lengthened in cancer patients with a higher expression level of MT1JP [44]. Functionally, overexpression of MT1JP was found to inhibit cell proliferation, migration and invasion, promote cell apoptosis in vitro and slow down tumor growth and metastasis in vivo [43,44]. Ding et al. reported low MT1L expression in bladder cancer [46]. These findings indirectly indicate the importance of our identified lncRNAs and pseudogenes for CNT-induced lung cancer.

LncRNAs and pseudogenes were shown to be involved in various cell processes by regulating the expression levels of mRNAs via ceRNA [34,35] or co-expression [36,37,39,47] mechanisms. Thus, in our study, we constructed ceRNA and co-expression networks to identify the interaction pairs of hub lncRNAs and pseudogenes. As a result, we found that MEG3 and RPL23AP64 acted as a ceRNA to positively regulate the expression levels of CPEB2, PHF21A and BAMBI by sponging miR-942-5p, and PVT1, ZNF826P, RPL23AP64 and TMEM198B regulated the expression levels of PSME3, SYNGAP1, SGK3 and CLU expression by absorbing miR-423-5p, miR-23a-3p and miR-15b-5p, respectively. Furthermore, MEG3 and ZNF826P directly modulated the transcription of ATM and SGK3; MT1L could co-express with ATM, SYNGAP1, CLU and SGK3; ARHGAP5-AS1 could co-express with TMED10 and NIP7; LINC00174 could co-express with NEDD4L; RPL23AP64 could co-express with SGK3; PVT1 could co-express with NIP7; and MT1JP could co-express with SGK3. Among these regulatory relationship pairs, only the binding of PVT1 and miR-423-5p was verified by dual-luciferase reporter, RNA immunoprecipitation and RNA pull-down assays [48]. The expression level of PVT1 was significantly and negatively correlated with miR-423-5p in cancer tissues [48]. Thus, other mechanisms may be the first evidence provided by our study. However, the expression levels and roles of related miRNAs or mRNAs had been reported previously in lung cancer or other cancers. For example, Dong et al. found that miR-942-5p was significantly upregulated in human LUAD tissues and cell lines [49]. Enforced expression of miR-942-5p significantly enhanced the growth [49], migration and invasion capacities of non-small cell lung cancer (NSCLC) cells [50]. GSE24279 dataset analysis and PCR experiments confirmed that miR-23a-3p was upregulated in pancreatic cancer compared with controls [51]. Analysis of NSCLC tissue data in TCGA database showed a higher expression level of miR-23a was significantly associated with a shorter OS rate [52]. Serum miR-15b-5p was detected to be significantly upregulated in NSCLCs. Multivariate logistic regression analysis revealed that miR-15b-5p expression was an independent diagnostic factor for the identification of patients with NSCLCs from controls [53]. Knockdown of miR-15b-5p restrained growth and invasiveness and induced apoptosis of breast and prostate cancer cells [54,55]. Knockout of CPEB2 was shown to promote oncogenic properties of MCF10A and MCF7 cells, including increased proliferation, migration, invasion, EMT and stem-like cell phenotypes. Injection of CPEB2-knockout MCF10A cells in mice resulted in the formation of subcutaneous tumors and spontaneous lung metastases [56]. Kaplan–Meier analyses of GSE4412 and GSE4271 datasets demonstrated that downregulation of PHF21A was closely associated with a decreased OS rate among patients with glioma. A multivariate analysis further confirmed that the expression level of PHF21A was an independent prognostic factor [57]. BAMBI is a negative regulator of the TGF-β signaling pathway. Downregulation of BAMBI was observed to promote TGF-β-induced EMT, migration and invasion of NSCLCs in vitro, along with increased tumor burdens and tumor growth in vivo [58]. Overexpression of BAMBI possessed antitumor potentialities [58,59]. Immunohistochemistry experiments showed that PSME3 was highly expressed in pancreatic cancer cells [60]. RNA interference-mediated silencing of PSME3 suppressed cancer cell proliferation, invasion and migration; induced cell apoptosis and cell cycle arrest at the G2/M phase; and enhanced radiosensitivity [61]. SYNGAP1 (also known as RASA5) was frequently methylated in multiple carcinomas to induce a decrease in its expression levels. Knockdown of RASA5 enhanced Ras signaling and then promoted tumor cell growth, migration and invasion via activation of EMT [62]. NSCLC patients with clusterin-positive tumors had a significantly longer survival period relative to those with clusterin-negative tumors (63% vs. 42%) [63]. Consistent with the tissues, the mRNA level of CLU was lower in lung cancer cell lines. Knockdown of CLU was verified to significantly promote the growth of lung cancer cells in vitro and in vivo [64]. A decreased expression level of ATM was associated with inferior OS and higher prostate cancer-specific mortalities [65]. Activation of the ATM/checkpoint kinase 2/p53-dependent signaling pathway was shown to inhibit the chemoresistance of HGC-27 cells to oxaliplatin [66]. The cell viability was significantly higher, but the apoptotic rate was significantly lower by transfection with TMED10 into a papillary thyroid cancer cell line than those in the control group [67]. NEDD4L was downregulated in NSCLCs, which was correlated with lymph node invasion, advanced stage and poor prognosis. Overexpression of NEDD4L significantly suppressed cell proliferation, migration and invasion abilities, whereas knockdown of NEDD4L enhanced the tumor metastasis of NSCLC cells [68]. NIP7 was significantly upregulated in the TCGA glioblastoma dataset [69]. In line with these studies, we also confirmed that CPEB2, PHF21A, BAMBI, ATM, SYNGAP1, CLU, NEDD4L and miR-423-5p were downregulated, whereas TMED10, PSME3, NIP7, miR-15b-5p and miR-23a-3p were upregulated in CNT-exposed lung cells and lung cancer samples. All of them (including miR-942) were associated with survival of lung cancer patients. Experimental studies revealed that SGK3 was an oncogenic gene [70,71]; however, TCGA analysis of several cancer data indicated SGK3 was downregulated compared with controls (including lung cancer, which was also confirmed in our prognosis and HPA analyses) (Figure S8). Thus, further experiments are necessary to confirm the expression levels and roles of SGK3 in CNT-related lung cancer. Furthermore, co-expression relationships between MT1JP and SYNGAP1/BAMBI/NEDD4L/ATM/CPEB2/CLU/PHF21A were present (all r > 0.6 and *p* < 0.01; Table S4), indicating MT1JP also functioned in lung cancer by influencing these genes.

To confirm whether our identified lncRNAs and pseudogenes were potential biomarkers for early diagnosis of CNT occupational workers with the risk to develop lung cancer, we performed ROC curve analysis based on the TCGA data of lung cancer. Our results showed the diagnostic accuracy of a four-lncRNA signature, a five-pseudogene signature or a nine-lncRNA-pseudogene signature were all higher than 80% (even approximate to 1), which was obviously higher than those of the 35-mRNA signature previously identified by Guo et al. (accuracy of 62–80%) [20] and essentially similar to a four-gene signature identified in our previous study [72]. Thus, we consider that our lncRNA and pseudogene signatures may be promising biomarkers for medical surveillance of lung cancer risk in occupational CNT-exposed workers.

To provide underlying preventive drugs, we mined the CTD database and selected some chemical molecules that reversed the expression levels of lncRNAs and pseudogenes. Our results showed that using supplements of *Schisandra chinensis* (containing a schizandrin A component), folic acid, zinc and gamma-linolenic Acid may be a potential approach to reduce the risk of developing lung cancer for populations with long-term CNT exposure. Our hypothesis can be indirectly validated because there studies have demonstrated the antitumor effects of these drugs. For example, Zhu et al. found schizandrin A treatment reduced viability, inhibited proliferation and induced cell cycle arrest and apoptosis of NSCLC cells [73]. Bae reported that higher foliate levels can decrease lung cancer risk in men (odds ratios = 0.82) and former smokers (odds ratios = 0.7) [74]. A meta-analysis performed by Charoenngam et al. showed that individuals with higher zinc intake had a significantly decreased risk of lung cancer, with a pooled risk ratio of 0.68 [75]. Dietary supplementation with gamma-linolenic acid was shown to inhibit the growth of a human lung carcinoma implanted in nude mice [76]. In vitro experiments also confirmed that gamma-linolenic acid was effective in inhibiting the migration and invasion of NSCLC cells [77].

This research has some limitations. First, this study preliminarily screened lncRNAs and pseudogenes associated with CNT-induced lung carcinogenesis based on two microarray data of human lung epithelial cells and the sequencing data of lung cancer patient tissues. Their expression and downstream function mechanisms (target genes identified by ceRNA or co-expression analysis) still needed further confirmation by more high-throughput datasets and wet experiments (including PCR, Western blot, overexpression or silencing of genes, dual-luciferase reporter assay, proliferation, apoptosis, invasion and metastasis analyses) in in vitro (different cell lines) and in vivo lung cancer models induced by different types of CNTs, especially for those first reported in our study, such as RPL23AP64, ZNF826P and TMEM198B. Second, CNT-induced lung cancer may be a long-term process. It may be difficult to collect samples from clinical populations. Thus, mice models with low-dose, chronic, long-term exposure to CNTs should be first established to observe the risk of development of lung cancer and validate the diagnostic values of our identified lncRNAs and pseudogenes. Furthermore, dietary supplementation of schizandrin A, folic acid, zinc or gamma-linolenic acid were also assigned for model mice to reveal their possible protective roles in the development of lung cancer in CNT occupational workers. However, a repository of biological samples from CNT-exposed workers needs to be created in the future by collaboration with other experts, which may be more beneficial to monitor biologically relevant changes of humans over time and to verify the effects of molecular biomarkers and drugs [78].

## 5. Conclusions

In the present study, we identified four lncRNAs (MEG3, ARHGAP5-AS1, LINC00174 and PVT1) and five pseudogenes (MT1JP, MT1L, RPL23AP64, ZNF826P and TMEM198B) that function as a ceRNA for miRNA–mRNA interactions or co-express with mRNAs as candidate biomarkers for surveillance of lung malignant transformation in occupational CNT-exposed workers. Dietary supplementation of schizandrin A, folic acid, zinc or gamma-linolenic acid may be a potential preventive strategy for CNT-induced lung cancer.

## Figures and Tables

**Table 1:** The expression levels of crucial genes.

		GSE41178 (SWCNT vs. Control)		GSE41178 (MWCNT vs. Control)	
		Log_2_FC	FDR	Log_2_FC	FDR
lncRNAs	ARHGAP5-AS1	1.46	3.91 × 10^4^	1.46	5.35 × 10^4^
	PVT1	0.7	1.04 × 10^3^	1.14	2.86 × 10^4^
	LINC00174	−1.23	3.31 × 10^4^	−1.01	2.14 × 10^3^
	MEG3	−1.09	1.44 × 10^2^	−1.37	2.00 × 10^3^
Pseudogenes	MT1L	−1.9	7.49 × 10^5^	−1.77	6.05 × 10^5^
	ZNF826P	−1.19	6.06 × 10^3^	−1.24	3.38 × 10^3^
	MT1JP	−0.87	5.15 × 10^4^	−1.21	2.86 × 10^4^
	RPL23AP64	−0.78	2.63 × 10^3^	−0.99	1.62 × 10^3^
	TMEM198B	−0.66	3.94 × 10^3^	−0.54	4.41 × 10^3^
mRNAs	NIP7	0.66	3.56 × 10^3^	0.75	8.43 × 10^4^
	TMED10	0.61	4.28 × 10^3^	0.61	6.93 × 10^3^
	PSME3	0.60	8.01 × 10^4^	0.57	5.52 × 10^3^
	CPEB2	−3.29	6.40× 10^5^	−3.25	1.63 × 10^4^
	CLU	−2.77	2.49 × 10^5^	−1.87	3.67 × 10^5^
	ATM	−2.26	3.58 × 10^4^	−1.7	3.58 × 10^4^
	SGK3	−2.04	3.89× 10^5^	−2.08	3.19 × 10^5^
	SYNGAP1	−1.45	6.28 × 10^4^	−1.08	1.50 × 10^3^
	BAMBI	−1.34	7.39 × 10^5^	−1.2	2.23 × 10^4^
	NEDD4L	−1.29	4.58 × 10^5^	−0.86	1.43 × 10^3^
	PHF21A	−1.15	9.71 × 10^5^	−0.79	6.65 × 10^3^

FC, fold change; FDR, false discovery rate; SWCNT, single-walled carbon nanotube; MWCNT, multiple-walled carbon nanotube.

**Table 2:** Topological characteristics of target genes regulated by lncRNAs in the PPI network.

Genes	DC	Genes	BC	Genes	CC	Genes	EC	Genes	LAC	Genes	IC	Genes	SC
MYC	89	MYC	69325.03	MYC	0.046924	MYC	0.345324	COL4A2	11.14286	MYC	4.519095	MYC	3.95 × 10^7^
ATM	53	CASP3	28914.29	CASP3	0.046573	HIST2H3PS2	0.232677	COL6A1	10.95652	ATM	4.37312	HIST2H3PS2	1.79 × 10^7^
HIST2H3PS2	49	ATM	26878.67	ATM	0.046346	CASP3	0.218635	COL11A1	10.88889	HIST2H3PS2	4.345062	CASP3	1.58 × 10^7^
CASP3	49	PPARG	21315.25	HIST2H3PS2	0.046318	ATM	0.213316	COL6A2	10.34783	CASP3	4.345062	ATM	1.51 × 10^7^
PPARG	40	HIST2H3PS2	17839.02	PPARG	0.046209	CCNB1	0.196486	COL5A2	10.2	PPARG	4.263963	CCNB1	1.28 × 10^7^
CCNB1	37	NRAS	16979.76	NRAS	0.046184	CHEK1	0.175156	COL1A1	9.67742	CCNB1	4.229326	CHEK1	1.02 × 10^7^
NRAS	36	PTK2	15259.33	CCNB1	0.046091	AURKA	0.154292	COL6A3	9.636364	NRAS	4.216698	AURKA	7896703
CHEK1	34	AURKA	15109.43	SMAD2	0.046085	FOS	0.145202	NID1	8.923077	CHEK1	4.189493	FOS	6983669
AURKA	33	SMAD2	14891.42	FOS	0.046038	CCND2	0.137003	CCNB1	8.648648	AURKA	4.17482	CCND2	6221439
SMAD2	32	HSPA9	12724.54	SP1	0.04601	NRAS	0.13518	LAMC1	8.6	SMAD2	4.159399	NRAS	6054038
FOS	31	TJP1	12550.71	SOD2	0.045982	CCNE2	0.135058	CHEK1	8.588235	COL1A1	4.14313	CCNE2	6049281
WDR5	31	TGFBR1	11898.11	PTK2	0.045967	PPARG	0.13326	LAMA4	8.5	FOS	4.14313	PPARG	5887511
COL1A1	31	UBE2I	11854.41	CHEK1	0.045967	RBBP4	0.12862	HIST2H3PS2	8.408163	WDR5	4.14313	RBBP4	5483687
RBBP4	30	RERE	11608.05	RBBP4	0.045964	LMNB1	0.124717	MYC	8.314607	PTK2	4.125951	LMNB1	5156968
PTK2	30	SOD2	11343.42	UBE2I	0.045952	SMAD2	0.121389	COL18A1	7.888889	RBBP4	4.125951	SMAD2	4895097
HSPA9	30	FOS	10771.87	AURKA	0.045945	SP1	0.117861	LEPREL1	7.818182	HSPA9	4.125951	SP1	4601198
UBE2I	29	TMED10	10569.32	CCNE2	0.045884	WDR5	0.112875	COL8A1	7.5	UBE2I	4.107765	WDR5	4226990
AGO2	27	RBBP4	10403.83	TGFBR1	0.045859	PLK4	0.111689	FBN1	7.428571	AGO2	4.068093	PLK4	4140534
CCNE2	26	DMD	10381.87	SIRT7	0.045847	RAD21	0.110684	COL11A2	7.375	CCNE2	4.046388	RAD21	4063987
NUP98	26	WDR5	10283.43	CCND2	0.045826	MCL1	0.110118	CCND2	7.363637	NUP98	4.046388	MCL1	4018060
TGFBR1	25	CHEK1	10165.99	AGO2	0.045816	NUP98	0.109068	CASP3	6.979592	TGFBR1	4.023259	NUP98	3947886
SP1	23	TFRC	9382.314	TJP1	0.045813	UBE2I	0.10781	CCNE2	6.923077	COL6A1	3.972192	UBE2I	3852734
SOD2	23	COL1A1	9344.245	RAD21	0.04581	TOP1	0.106882	NUP133	6.833334	COL6A2	3.972192	TOP1	3787653
TJP1	23	CCNB1	9142.68	WDR5	0.045786	AGO2	0.104175	RAD51AP1	6.714286	FH	3.972192	AGO2	3599771
NCBP2	23	TXNRD1	8277.166	HSPA9	0.04578	ATAD2	0.098454	AURKA	6.606061	ACLY	3.972192	PTK2	3242236
FH	23	PPP6C	7909.077	AKT3	0.04578	PTK2	0.097769	PLK4	6.454546	SP1	3.972192	ATAD2	3214702
ACLY	23	SP1	7895.044	FGFR2	0.045773	DTL	0.095207	NDC1	6.428571	SOD2	3.972192	DTL	3011290
COL6A1	23	FGFR2	7834.322	NUP98	0.045773	RNF2	0.091295	ATM	6.415094	NCBP2	3.972192	RNF2	2763911
COL6A2	23	CCT3	7686.299	PGK1	0.045767	CDC27	0.089433	MCL1	6.375	TJP1	3.972192	CDC27	2653227
CCND2	22	CCNE2	7528.156	TOP1	0.045758	MYBL2	0.0881	ATAD2	6.375	COL6A3	3.943907	MYBL2	2572502
PLK4	22	ITCH	7308.306	PRKCD	0.045749	KAT6A	0.087586	DTL	6.363637	CCND2	3.943907	KAT6A	2543617
DTL	22	CSF2	7290.804	LMNB1	0.045749	AKT3	0.081991	LEPREL2	6.285714	PLK4	3.943907	AKT3	2227275
PGK1	22	PINK1	7266.99	MCL1	0.045734	PAX6	0.080551	LMNB1	6.210527	DTL	3.943907	COL1A1	2210530
COL6A3	22	AKT3	7194.102	RUNX1	0.045715	SIRT7	0.080551	ITGA7	6.166667	PGK1	3.943907	SIRT7	2151882
CCT3	22	AGO2	6931.627	EPAS1	0.045691	AGO1	0.080408	NUP50	6.117647	CCT3	3.943907	PAX6	2149573
TOP1	21	UBQLN1	6730.845	PAX6	0.045688	RCOR1	0.07753	NUP98	6.076923	COL4A2	3.91349	AGO1	2144101
RNF2	21	ACLY	6575.635	RNF2	0.045679	PTCH1	0.077235	HK2	6	TOP1	3.91349	RCOR1	1993021
AGO1	21	GOLPH3	6248.143	AGO1	0.045664	COL1A1	0.076574	FH	6	RNF2	3.91349	PTCH1	1976036
COL4A2	21	PLK4	6190.994	PTCH1	0.045651	SOD2	0.076143	SERPINH1	5.888889	AGO1	3.91349	SOD2	1923597
ABCE1	21	SERPINH1	6138.03	PAK2	0.045648	TGFBR1	0.075727	CS	5.75	ABCE1	3.91349	TGFBR1	1910805
RAD21	20	MAPRE3	6080.12	YES1	0.045648	RUNX1	0.075226	GPI	5.733333	COL5A2	3.880701	RUNX1	1875071
AKT3	20	EPAS1	5907.968	KAT6A	0.045639	RAD51AP1	0.073014	PGK1	5.727273	LAMC1	3.880701	RAD51AP1	1771838
EPAS1	20	PAK2	5893.081	COL1A1	0.045636	CCNH	0.072743	MYBL2	5.571429	RAD21	3.880701	CCNH	1754899
ITCH	20	SRPR	5857.721	PLK4	0.045609	FGFR2	0.070863	ACLY	5.565218	DMD	3.880701	FGFR2	1663734
LAMC1	20	ABCE1	5784.403	MAX	0.045606	PGK1	0.070127	FOS	5.548387	TXNRD1	3.880701	PGK1	1635160
DMD	20	PPL	5654.79	CDC27	0.045603	EPAS1	0.069382	PTK2	5.533333	AKT3	3.880701	EPAS1	1595363
COL5A2	20	BCL2	5523.898	ITCH	0.045597	TJP1	0.068124	SEH1L	5.466667	EPAS1	3.880701	TJP1	1540930
TXNRD1	20	RAD21	5511.951	TFRC	0.045591	CSF2	0.068085	SMAD2	5.4375	ITCH	3.880701	CSF2	1535941
TMED10	20	PGK1	5500.145	MYBL2	0.045579	PRKCD	0.066951	AGO2	5.333334	TMED10	3.880701	PRKCD	1485477
LMNB1	19	SLC25A33	5496.237	PSME3	0.045572	MAX	0.065213	NIP7	5.333334	LMNB1	3.845279	TGFB3	1411799
FGFR2	19	NEDD4L	5484.699	TGFB3	0.045566	TGFB3	0.064884	TRMT11	5.230769	NEDD4L	3.845279	MAX	1409118
NEDD4L	19	CCNH	5324.429	ACLY	0.04556	NCBP2	0.064605	SP1	5.217392	FGFR2	3.845279	NCBP2	1385477
RUNX1	18	F11R	5165.416	NCBP2	0.045551	HSPA9	0.064468	RCOR1	5.142857	COL11A1	3.806871	HSPA9	1381436
CCNH	18	FH	5138.533	ATAD2	0.045548	HK2	0.0622	NRAS	5.111111	COL18A1	3.806871	HK2	1286048
DDX18	18	AGO1	5105.469	NEDD4L	0.045542	HIST1H3J	0.061885	RAD21	5.1	SERPINH1	3.806871	HIST1H3J	1270916
COL18A1	18	DDX18	5075.202	GOLPH3	0.045539	NFYA	0.06188	PNO1	5.058824	DDX18	3.806871	NFYA	1268920
COL11A1	18	SIRT7	5028.431	HK2	0.045539	NDC1	0.061369	RBBP4	5	CCNH	3.806871	NDC1	1251614
SERPINH1	18	NCBP2	4760.606	CCNH	0.045527	MDM4	0.060536	TGFB3	5	RUNX1	3.806871	MDM4	1214375
CDC27	17	TCEA1	4649.523	CSF2	0.045521	PAK2	0.06015	NOC3L	5	NUP50	3.765087	PAK2	1205645
KAT6A	17	CCND2	4621.744	RCOR1	0.045521	POLE3	0.060104	RBM19	5	HK2	3.765087	POLE3	1200180

DC, degree centrality; BC, betweenness centrality; CC, closeness centrality; EC, eigenvector centrality; LAC, local average connectivity; IC, information centrality; SC, subgragh centrality; PPI, protein–protein interaction.

**Table 3:** Topological characteristics of target genes regulated by pseudogenes in the PPI network.

Genes	DC	Genes	BC	Genes	CC	Genes	EC	Genes	LAC	Genes	IC	Genes	SC
HIST2H3PS2	24	IGF1	12,657.02	IGF1	0.020131	HIST2H3PS2	0.336233	HIST2H2AB	8.666667	HIST2H3PS2	2.791786	HIST2H3PS2	15879.49
ATM	23	ATM	10,286.39	ATM	0.020117	HIST1H4F	0.293143	HIST2H3D	8.533334	ATM	2.778953	HIST1H4F	12090.06
IGF1	21	ICAM1	8398.269	NFKBIA	0.020094	HIST3H3	0.282137	HIST1H1E	8.4	IGF1	2.750203	HIST3H3	11213.9
HIST1H4F	18	NFKBIA	6650.969	HIST2H3PS2	0.020077	HIST2H3D	0.273608	HIST1H3J	8.307693	HIST1H4F	2.697331	HIST2H3D	10568.27
CCL2	18	HIST2H3PS2	5622.567	ICAM1	0.020076	HIST1H2AJ	0.269709	HIST3H3	8.125	CCL2	2.697331	HIST1H2AJ	10266.41
NFKBIA	17	CCL2	5539.708	IKBKB	0.020066	HIST2H2AB	0.2494	HIST1H2AI	8	NFKBIA	2.676373	HIST2H2AB	8764.285
HIST3H3	16	TUBB4A	5141.43	SOD2	0.020055	HIST1H3J	0.247382	HIST1H2AJ	7.625	HIST3H3	2.65333	HIST1H3J	8645.107
HIST1H2AJ	16	IKBKB	5052.972	CCL2	0.020028	ATM	0.233653	HIST1H4F	7.555555	HIST1H2AJ	2.65333	ATM	7783.067
HIST2H3D	15	RCVRN	4885.731	PAX6	0.020013	HIST1H2AI	0.220319	HIST1H1A	7.4	HIST2H3D	2.627873	HIST1H2AI	6837.918
POU5F1	14	SOD2	4803.247	POU5F1	0.020007	HIST1H1E	0.217499	HIST2H3PS2	6.75	POU5F1	2.599608	HIST1H1E	6684.745
ICAM1	14	AKT3	4681.91	TUBB4A	0.020004	HIST1H1A	0.209097	HIST1H2AD	5.333334	ICAM1	2.599608	HIST1H1A	6168.202
HIST1H3J	13	CCND2	4486.447	AKT3	0.020001	HIST1H2AD	0.19763	PHF21A	5.333334	HIST1H3J	2.568042	HIST1H2AD	5525.728
IKBKB	13	CD59	4309.046	CSF2	0.019999	PHF21A	0.176246	TNFAIP3	4.25	IKBKB	2.568042	PHF21A	4369.904
HIST2H2AB	12	POU5F1	3915.681	CCND2	0.019997	WHSC1	0.160865	WHSC1	4	HIST2H2AB	2.532561	WHSC1	3640.597
HIST1H2AD	12	WWC1	3888.129	FGFR1	0.019978	KAT6A	0.150582	IKBKB	3.846154	HIST1H2AD	2.532561	KAT6A	3188.971
PAX6	11	CDK5	3827.028	FGFR2	0.019959	CBX6	0.137474	ATM	3.826087	PAX6	2.492376	CBX6	2673.861
SOD2	11	CSF2	3700.629	WWC1	0.019931	IGF1	0.112766	KAT6A	3.75	SOD2	2.492376	IGF1	2112.217
CSF2	11	KIF5C	3555.034	HIST1H4F	0.01993	POU5F1	0.102878	CBX6	3.5	CSF2	2.492376	POU5F1	1541.507
RCVRN	11	PAX6	3298.453	RCVRN	0.019922	PAX6	0.100751	CCL2	3.333333	RCVRN	2.492376	PAX6	1454.034
HIST1H2AI	10	WWOX	2962.674	TNFAIP3	0.019909	AIRE	0.096106	TUBB8	3.2	HIST1H2AI	2.446504	NFKBIA	1431.716
HIST1H1E	10	ZNF395	2778.826	WWOX	0.019906	CCND2	0.089509	NFKBIA	3.058824	HIST1H1E	2.446504	IKBKB	1324.191
HIST1H1A	10	TGM1	2707.593	TUBA1A	0.019905	NFKBIA	0.087309	AIRE	3	HIST1H1A	2.446504	AIRE	1304.235
CCND2	10	SYN3	2563.518	CDK5	0.01989	IKBKB	0.084984	SOD2	2.909091	CCND2	2.446504	CCND2	1161.27
TUBB4A	10	GNG12	2428.181	BCL2	0.019888	CTBP1	0.074958	ICAM1	2.857143	TUBB4A	2.446504	CTBP1	804.1904
PHF21A	9	FZR1	2387.046	SIRPA	0.01988	TET3	0.06408	TUBA1A	2.857143	PHF21A	2.393643	CCL2	775.7595
WHSC1	9	CAMK1G	2352.95	CTBP1	0.019877	AKT3	0.059664	TUBA4A	2.666667	WHSC1	2.393643	SOD2	677.2321
AKT3	9	CAV3	2350.628	KAT6A	0.019877	FGFR2	0.058941	IGF1	2.571429	AKT3	2.393643	ICAM1	610.6983
KAT6A	8	MYO5B	2350.244	ZC3H12A	0.019873	SOD2	0.056252	TUBB4A	2.4	KAT6A	2.332049	TET3	582.8483
CBX6	8	XCL1	2339.667	HIST3H3	0.019869	EYA2	0.052028	VAMP1	2.4	CBX6	2.332049	AKT3	560.5861
FGFR2	8	EXOC4	2334.192	THPO	0.019866	WWC1	0.050389	CSF2	2.363636	FGFR2	2.332049	FGFR2	529.6174
FGFR1	8	COPZ2	1880	EYA2	0.019866	TAL1	0.048814	CCND2	2	FGFR1	2.332049	CSF2	405.3018
TNFAIP3	8	KCND3	1875.697	WHSC1	0.019865	CCL2	0.048485	ZC3H12A	2	TNFAIP3	2.332049	EYA2	384.0464
CDK5	8	CCK	1872	PHF21A	0.019863	FGFR1	0.047015	SIRPA	2	CDK5	2.332049	WWC1	368.5083
KIF5C	8	LIN7C	1762.686	IGFBP3	0.019861	TNRC6C	0.046971	NFKBIE	2	KIF5C	2.332049	FGFR1	354.2804
VAMP8	8	FGFR1	1600.013	HIST2H2AB	0.019858	ICAM1	0.044627	TNIP2	2	VAMP8	2.332049	TNFAIP3	343.6676
CTBP1	7	RAB11FIP5	1546.701	HIST1H2AI	0.019855	ERCC6	0.044454	SYT17	2	CTBP1	2.259382	TAL1	341.3784
WWC1	7	F11R	1532.283	HOXB7	0.019852	TUBA1A	0.03925	LCE1D	2	WWC1	2.259382	TNRC6C	314.0037
TUBA1A	7	F7	1457.162	ADRBK1	0.019851	CSF2	0.037267	LCE1F	2	TUBA1A	2.259382	ERCC6	280.5435
WWOX	7	UBE2K	1414	HIST1H2AJ	0.019841	TNFAIP3	0.032499	POU5F1	1.857143	WWOX	2.259382	TUBA1A	252.0046
CD59	7	TCEAL6	1411	CLU	0.01984	CDK5	0.030225	FGFR2	1.75	GNG12	2.259382	ZC3H12A	188.933
GNG12	7	WDFY2	1410	ERCC6	0.019832	WWOX	0.02852	FGFR1	1.75	CD59	2.259382	TUBB4A	185.0031
CAV3	7	CREB3L3	1407.115	HIST1H1A	0.019829	TUBB4A	0.02729	VAMP8	1.75	CAV3	2.259382	CDK5	147.8962
ZC3H12A	6	BID	1394.544	TET3	0.019826	ZC3H12A	0.027068	CTBP1	1.714286	ZC3H12A	2.172348	WWOX	136.4548
BID	6	RGMA	1341.624	CD59	0.019821	FOXP1	0.02444	SYN3	1.666667	BID	2.172348	SIRPA	98.61929
BCL2	6	CACNA1E	1306.522	CADM1	0.019819	TACC3	0.02356	AKT3	1.555556	BCL2	2.172348	FOXP1	96.34972
TUBA4A	6	SOD3	1285.36	NRG1	0.019818	BID	0.0221	TET3	1.5	TUBA4A	2.172348	RCVRN	90.32636
SYN3	6	VAMP8	1285.289	HIST2H3D	0.019818	FGFRL1	0.01855	IGFBP3	1.5	EXOC4	2.172348	TACC3	86.21384
EXOC4	6	TUBA1A	1254.487	CSH1	0.019812	SIRPA	0.01763	KCND3	1.5	SYN3	2.172348	NFKBIE	83.78503
TGM1	6	CLU	1220.285	HIST1H3J	0.019812	RCVRN	0.017589	PKP2	1.5	TGM1	2.172348	TNIP2	83.78503
TAL1	5	BCL2	1190.642	BID	0.019811	BCL2	0.017578	TUBA3C	1.5	FOXP1	2.066228	BID	81.1344
FOXP1	5	FNDC5	1174.097	FNDC5	0.01981	ZIC1	0.017441	PPL	1.5	F7	2.066228	IGFBP3	69.96769
TUBB8	5	SPTB	1171.549	SGK3	0.01981	NFKBIE	0.017346	PAX6	1.454546	VAMP1	2.066228	BCL2	66.91744
FZR1	5	ZIC1	1159.743	HAND2	0.019808	TNIP2	0.017346	RCVRN	1.454546	TAL1	2.066228	FGFRL1	51.49969
CLU	5	SYNGAP1	1106.794	IGFBP5	0.019805	HOXB7	0.016011	BCL2	1.333333	FZR1	2.066228	THPO	51.38118
VAMP1	5	PLAT	1016.892	PLAT	0.019797	IGFBP3	0.015366	PTX3	1.333333	CLU	2.066228	ZIC1	50.78407
SYT11	5	CTBP1	992.8071	HIST1H2AD	0.019796	THPO	0.013824	RGMB	1.333333	TUBB8	2.066228	CADM1	50.12931
SCN1B	5	CABP4	974.4585	LIN7C	0.019793	NRG1	0.013609	TCEAL2	1.333333	SYT11	2.066228	HOXB7	46.41033
F7	5	SCN1B	959.0853	NFKBIE	0.019793	FZR1	0.012277	FOXP1	1.2	CACNA1E	2.066228	PTX3	45.3798
CACNA1E	5	NLGN2	958.3921	TNIP2	0.019793	HAND2	0.011935	SYT11	1.2	SCN1B	2.066228	TUBA4A	40.56611
AIRE	4	CBX6	949.5727	TUBA4A	0.01979	PCGF3	0.011594	SCN1B	1.2	AIRE	1.933961	PLAT	39.07808

DC, degree centrality; BC, betweenness centrality; CC, closeness centrality; EC, eigenvector centrality; LAC, local average connectivity; IC, information centrality; SC, subgragh centrality; PPI, protein–protein interaction.

**Table 4:** Diagnostic values of our identified lncRNAs and pseudogenes.

		AUC	Sensitivity (%)	Specificity (%)
LUAD	ARHGAP5-AS1	0.595	30.65	100.00
	LINC00174	0.733	53.64	83.05
	MEG3	0.620	40.75	84.75
	PVT1	0.974	88.97	94.42
	MEG3 + ARHGAP5-AS1 + LINC00174 + PVT1	0.976	93.83	91.53
	MT1JP	0.855	77.57	84.75
	MT1L	0.743	57.76	96.61
	RPL23AP64	0.512	28.79	79.66
	TMEM198B	0.579	36.26	88.14
	ZNF826P	0.519	40.56	91.53
	MT1L + MT1JP + RPL23AP64 + TMEM198B + ZNF826P	0.834	89.35	66.10
	All lncRNAs+all pseudogenes	0.977	94.39	89.93
LUSC	ARHGAP5-AS1	0.768	54.18	93.88
	LINC00174	0.768	58.37	85.71
	MEG3	0.525	46.61	71.43
	PVT1	0.975	95.22	91.84
	MEG3+ARHGAP5-AS1+LINC00174+PVT1	0.990	93.82	95.92
	MT1JP	0.939	84.06	93.88
	MT1L	0.795	65.94	95.92
	RPL23AP64	0.721	66.53	69.39
	TMEM198B	0.541	25.50	95.92
	ZNF826P	0.765	66.33	91.84
	MT1L + MT1JP + RPL23AP64 + TMEM198B + ZNF826P	0.925	86.06	87.76
	All lncRNAs+all pseudogenes	0.994	95.42	97.96

LUAD, lung adenocarcinoma; LUSC, lung squamous cell carcinoma; AUC, the area under the receiver operating characteristic curve.

## Data Availability

All data were downloaded from GEO (GSE41178 and GSE56104; http://www.ncbi.nlm.nih.gov/geo/, accessed on 5 September 2021) and TCGA-LUAD/LUSC (https://portal.gdc.cancer.gov/, accessed on 5 September 2021) databases.

## Supplementary Materials

The following are available online at https://www.mdpi.com/article/10.3390/ijerph19052936/s1, Figure S1: Venn diagram to show the overlapped genes. (A) commonly upregulated DE-lncRNAs, DE-pseudogenes and DE-mRNAs between SWCNT and MWCNT; (B) commonly downregulated lncRNAs, pseudogenes and mRNAs between SWCNT and MWCNT; (C) the overlap between module lncRNAs/pseudogenes and DE-lncRNAs/DE-pseudogenes. DE-lncRNAs, differentially expressed lncRNAs; DE-pseudogenes, differentially expressed pseudogenes; DE-mRNAs, differentially expressed mRNAs. Figure S2: GEPIA analysis to validate the expression of mRNAs in lung cancer. N = 50 or 59 indicates the control is match TCGA normal data; N = 338 or 347 indicates the control is match TCGA normal and GTEx data; asterisk (*) indicates the statistical significance at the threshold of |log_2_FC| > 0.5 and *p*-value < 0.05. Figure S3: UALCAN analysis to validate the expression of mRNAs in lung adenocarcinoma. Proteomic data from Clinical Proteomic Tumor Analysis Consortium (CPTAC) are used. Figure S4: HPA immunohistochemical staining results to validate the expression of mRNAs in lung cancer. LUAD, lung adenocarcinoma; LUSC, and squamous cell carcinomas. Figure S5: Validation of the prognosis of mRNAs in lung cancer. (A–G) PHF21A (A), TMED10 (B), NEDD4L (C), NIP7 (D), SYNGAP1 (E), CLU (F), SGK3 (G). Prediction for overall survival of LUAD patients by analysis of TCGA data; (H-J) CPEB2 (H), BAMBI (I), ATM (J). Prediction for overall survival of all lung cancer patients by analysis of chip data. HR, hazard ratio; LUAD, lung adenocarcinoma; TCGA, The Cancer Genome Atlas. Figure S6: Validation of the expression and prognosis of miR-942, miR-23a, miR-376b and miR-15b. A-D: the expression of miRNAs; E-H, the prognosis of miRNAs. LUAD, lung adenocarcinoma; LUSC, and squamous cell carcinomas. Figure S7: Validation of the expression and prognosis of miR-423 and its target mRNA PSME3. A: the prognosis of miR-423; B: the expression of PSME3; C: the prognosis of PSME3 for overall survival of TCGA LUAD samples; D: the prognosis of PSME3 for recurrence-free survival of TCGA LUAD samples; E: the prognosis of PSME3 for recurrence-free survival of TCGA LUSC samples. LUAD, lung adenocarcinoma; LUSC, and squamous cell carcinomas; TCGA, The Cancer Genome Atlas. Figure S8: SGK3 downregulated in several cancers. Asterisk indicates the statistical significance at the threshold of |log_2_FC| > 0.5 and *p*-value < 0.05. CESC, cervical squamous cell carcinoma and endocervical adenocarcinoma; OV, Ovarian serous cystadenocarcinoma; UCEC, uterine corpus endometrial carcinoma; UCS, uterine carcinosarcoma. Table S1: Survival analysis for lncRNAs. Table S2: The ceRNA interaction pairs for lncRNAs and pseudogenes. Table S3: The co-expression interaction pairs for lncRNAs and pseudogenes. Table S4: The protein-protein interaction pairs for lncRNAs and pseudogenes. Table S5: Survival analysis for mRNAs. Table S6: Function enrichment results for all genes in the protein-protein interaction networks of lncRNAs and pseudogenes.
