# Supplementary material for: Identification of Candidate lncRNA and Pseudogene Biomarkers Associated with Carbon-Nanotube-Induced Malignant Transformation of Lung Cells and Prediction of Potential Preventive Drugs
Source: Int J Environ Res Public Health. 2022 Mar 2;19(5):2936. doi: 10.3390/ijerph19052936 (PMC8910615; doi:10.3390/ijerph19052936)
Supplement: Supplementary file 1 [file ijerph-19-02936-s001.zip › Table S1.pdf]

**Table S1 Survival analysis for lncRNAs**

| | | | | HR | 95%CI | P-value |
|---|---|---|---|---|---|---|
| TCGA | OS | LUAD | LINC00174 | 0.74 | 0.55-0.99 | 0.044 |
| | RFS | LUAD | LOC644656 | 1.66 | 1.08-2.55 | 0.02 |
| | | LUSC | MEG3 | 0.6 | 0.36-0.99 | 0.042 |
| | | | LINC00863 | 0.5 | 0.27-0.93 | 0.025 |
| | | | LINC00174 | 0.49 | 0.29-0.82 | 0.0062 |
| | | | WDFY3-AS2 | 0.52 | 0.31-0.87 | 0.011 |
| Chip | OS | LUAD | 210794_s_at (MEG3) | 0.66 | 0.53-0.84 | 6.4E-04 |
| | | | 1558290_a_at (PVT1) | 1.54 | 1.2-1.97 | 6.4E-04 |
| | | | 1562953_s_at (WDFY3-AS2) | 0.51 | 0.4-0.64 | 1.6E-08 |
| | | LUSC | 1558290_a_at (PVT1) | 1.61 | 1.17-2.22 | 0.0033 |
| | | All | 210794_s_at (MEG3) | 0.81 | 0.71-0.92 | 9.7E-04 |
| | | | 1558290_a_at (PVT1) | 1.58 | 1.34-1.87 | 3.3E-08 |
| | | | 1562953_s_at (WDFY3-AS2) | 0.54 | 0.46-0.65 | 9.6E-13 |
| | FPS | LUAD | 228889_at (ARHGAP5-AS1) | 1.44 | 1.04-1.98 | 0.027 |
| | | | 210794_s_at (MEG3) | 0.68 | 0.49-0.95 | 0.024 |
| | | | 1558290_a_at (PVT1) | 1.69 | 1.21-2.36 | 0.0016 |
| | | | 1562953_s_at (WDFY3-AS2) | 0.52 | 0.37-0.71 | 4.9E-05 |
| | | LUSC | 228889_at (ARHGAP5-AS1) | 1.88 | 1.12-3.18 | 0.016 |
| | | | 1558290_a_at (PVT1) | 1.83 | 1.02-3.31 | 0.04 |
| | | All | 228889_at (ARHGAP5-AS1) | 1.55 | 1.17-2.05 | 1.9E-03 |
| | | | 1558290_a_at (PVT1) | 1.73 | 1.32-2.26 | 6.1E-05 |
| | | | 1562953_s_at (WDFY3-AS2) | 0.64 | 0.48-0.85 | 1.9E-03 |
| | PFS | LUAD | 210794_s_at (MEG3) | 0.59 | 0.35-0.99 | 0.042 |
| | | | 1558290_a_at (PVT1) | 1.72 | 1.02-2.9 | 0.038 |
| | | All | 1558290_a_at (PVT1) | 1.76 | 1.13-2.72 | 0.011 |

OS, overall survival; RFS, recurrence-free survival; FPS, first progression survival; PPS, post progression survival; HR, hazard ratio; CIs, confidence intervals; LUAD, lung adenocarcinoma; LUSC, and squamous cell carcinomas; TCGA, The Cancer Genome Atlas.
