# Supplementary material for: Identification of Candidate lncRNA and Pseudogene Biomarkers Associated with Carbon-Nanotube-Induced Malignant Transformation of Lung Cells and Prediction of Potential Preventive Drugs
Source: Int J Environ Res Public Health. 2022 Mar 2;19(5):2936. doi: 10.3390/ijerph19052936 (PMC8910615; doi:10.3390/ijerph19052936)
Supplement: Supplementary file 1 [file ijerph-19-02936-s001.zip › Table S5.pdf]

Table S5 Survival analysis for mRNAs

				HR	95%CI	P-value
TCGA	OS	LUAD	PHF21A	0.73	0.54-0.98	0.034
			TMED10	1.36	1.01-1.81	0.039
			NEDD4L	0.66	0.49-0.89	0.006
			NIP7	1.47	1.09-1.98	0.012
			SYNGAP1	0.64	0.48-0.86	0.0028
			CLU	0.73	0.54-0.99	0.042
			SGK3	0.58	0.43-0.77	0.00021
		LUSC	PHF21A	0.72	0.54-0.96	0.026
	RFS	LUAD	CLU	0.6	0.4-0.92	0.017
Chip	OS	All	CPEB2	0.45	0.38-0.54	1E-16
			PHF21A	0.75	0.63-0.89	0.0012
			BAMBI	0.83	0.73-0.95	0.0074
			ATM	0.55	0.47-0.65	3.4E-13
			NEDD4L	0.53	0.43-0.66	3.4E-09
			CLU	0.44	0.37-0.53	1E-16
	FPS		CPEB2	0.49	0.36-0.67	4.5E-06
			ATM	0.51	0.4-0.65	5.9e-08
			NEDD4L	0.37	0.25-0.54	1E-07
			CLU	0.47	0.38-0.59	6.7E-12
			SGK3	0.63	0.51-0.77	7.9E-06
	PPS		CPEB2	0.46	0.3-0.71	0.00028
			ATM	0.64	0.47-0.87	0.0042
			NEDD4L	0.62	0.4-0.96	0.031
			CLU	0.54	0.41-0.7	2.3E-06
	OS	LUAD	CPEB2	0.31	0.23-0.42	1.5E-15
			PHF21A	0.71	0.56-0.91	0.0055
			BAMBI	0.71	0.55-0.91	0.0058
			ATM	0.31	0.22-0.43	3.6E-014
			TMED10	1.33	1.05-1.68	0.016
			NEDD4L	0.46	0.35-0.61	2E-08
			CLU	0.3	0.22-0.39	1E-16
	FPS		CPEB2	0.46	0.33-0.65	6.5E-06
			PHF21A	0.7	0.51-0.96	0.028
			ATM	0.44	0.3-0.63	4.1E-06
			TMED10	1.57	1.15-2.14	0.0044
			NEDD4L	0.3	0.2-0.46	4.4E-09
			CLU	0.43	0.31-0.59	5.6E-08
			SGK3	0.58	0.42-0.79	0.00049
	PPS		CPEB2	0.54	0.32-0.91	0.018
			ATM	0.54	0.33-0.87	0.01
			CLU	0.55	0.33-0.91	0.018

	OS	LUSC	SGK3	0.74	0.56-0.96	0.024
	FPS		NIP7	1.75	1.02-3	0.041
			CLU	0.49	0.28-0.84	0.0076

OS, overall survival; RFS, recurrence-free survival; FPS, first progression survival; PPS, post progression survival; HR, hazard ratio; CIs, confidence intervals; LUAD, lung adenocarcinoma; LUSC, and squamous cell carcinomas; TCGA, The Cancer Genome Atlas.
