# Supplementary figures and images for: Identification of Candidate lncRNA and Pseudogene Biomarkers Associated with Carbon-Nanotube-Induced Malignant Transformation of Lung Cells and Prediction of Potential Preventive Drugs
Source: Int J Environ Res Public Health. 2022 Mar 2;19(5):2936. doi: 10.3390/ijerph19052936 (PMC8910615; doi:10.3390/ijerph19052936)

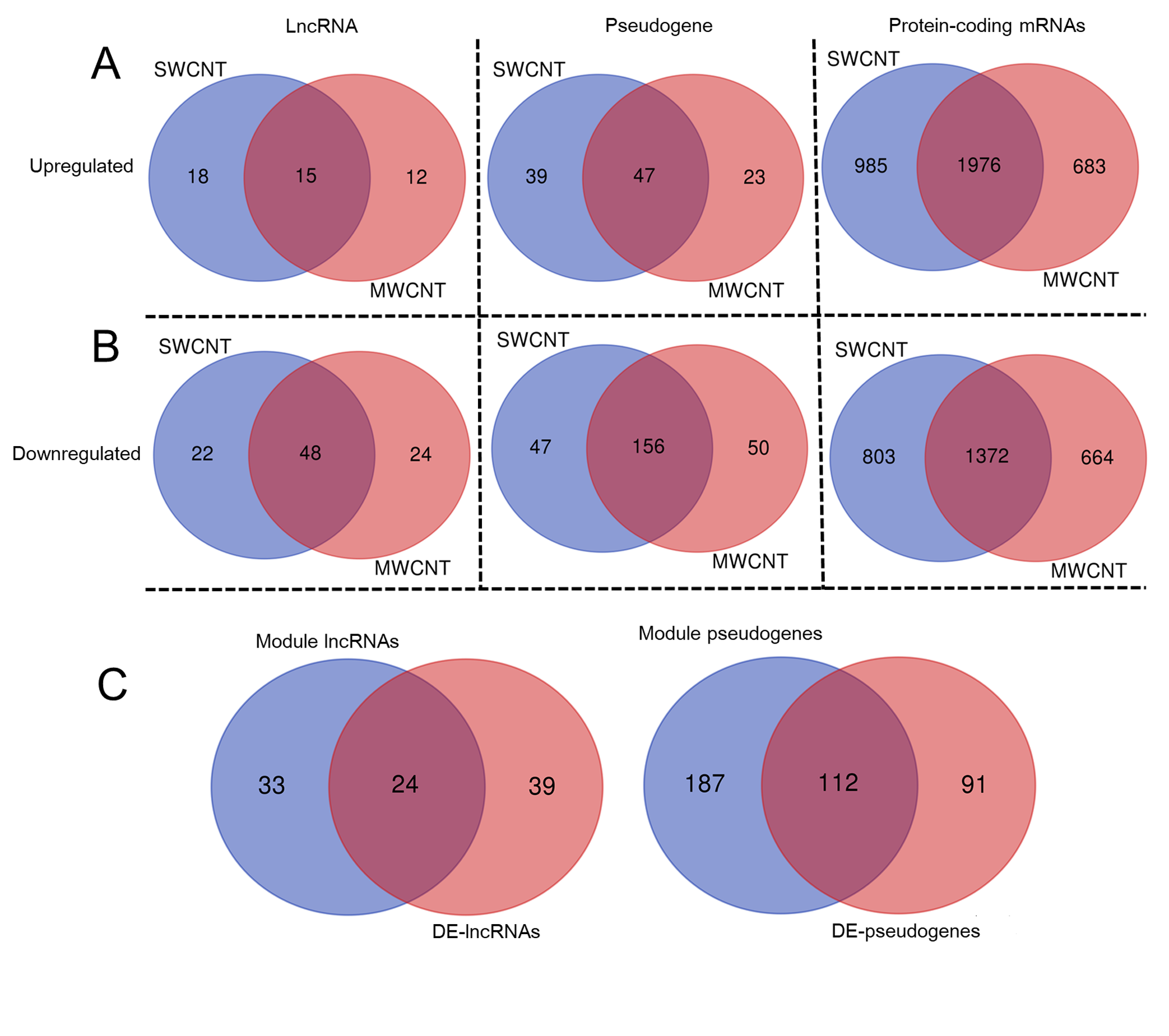

Supplement: Supplementary file 1 [file ijerph-19-02936-s001.zip › Figure S1.tif]

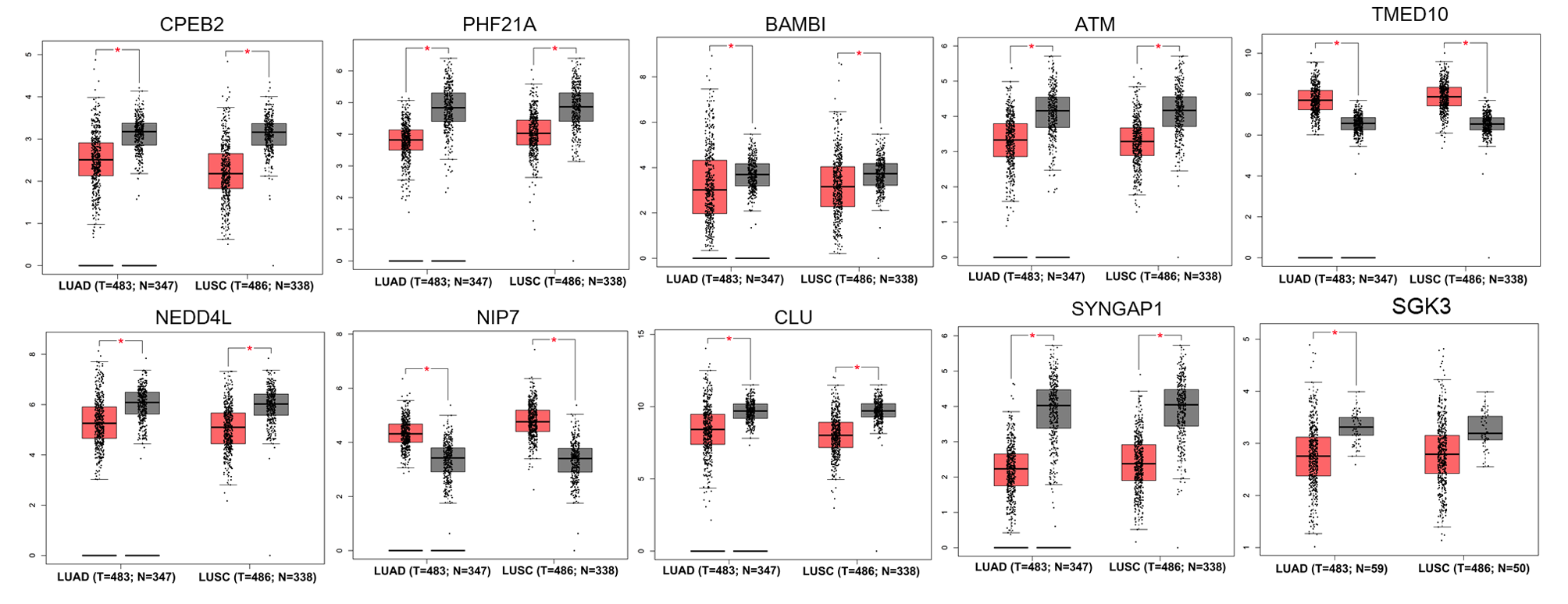

Supplement: Supplementary file 1 [file ijerph-19-02936-s001.zip › Figure S2.tif]

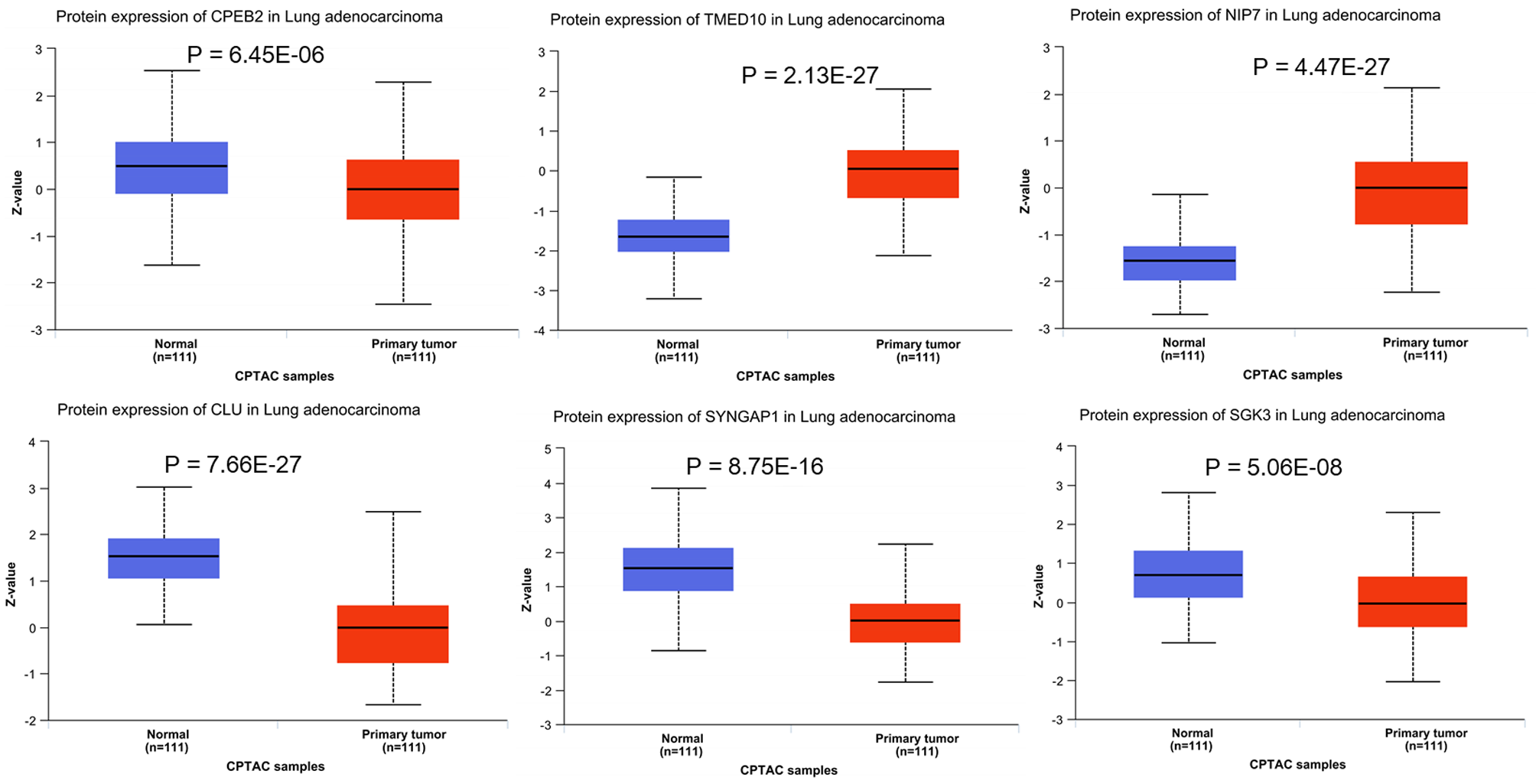

Supplement: Supplementary file 1 [file ijerph-19-02936-s001.zip › Figure S3.tif]

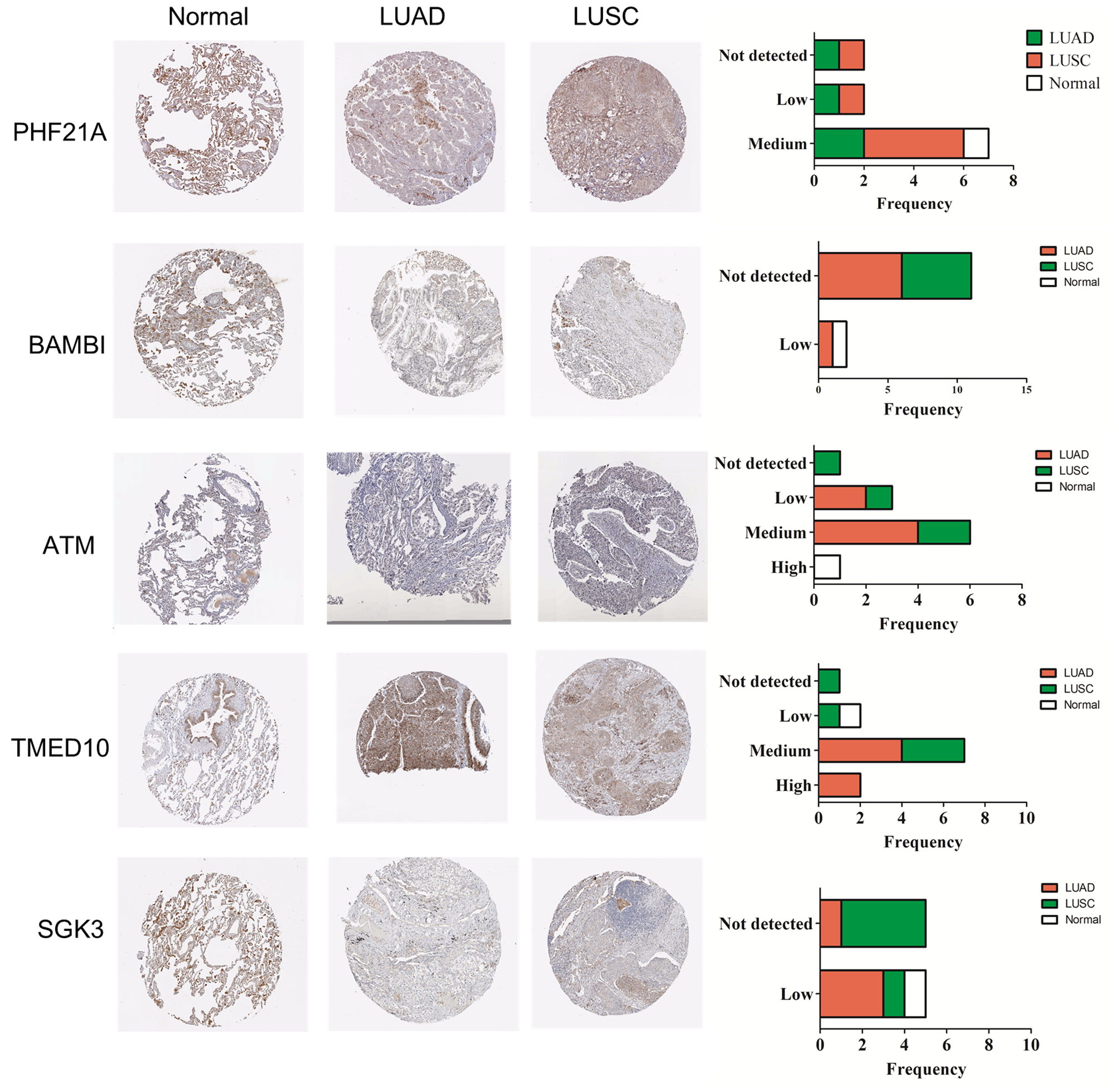

Supplement: Supplementary file 1 [file ijerph-19-02936-s001.zip › Figure S4.tif]

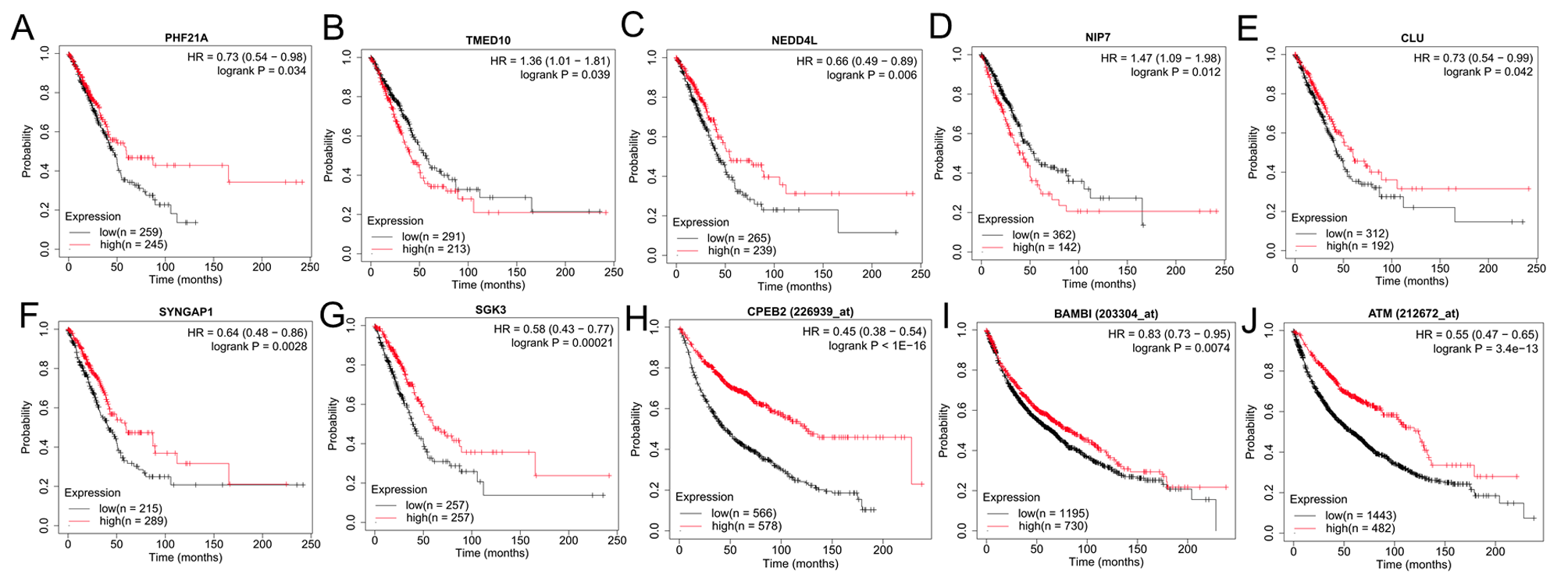

Supplement: Supplementary file 1 [file ijerph-19-02936-s001.zip › Figure S5.tif]

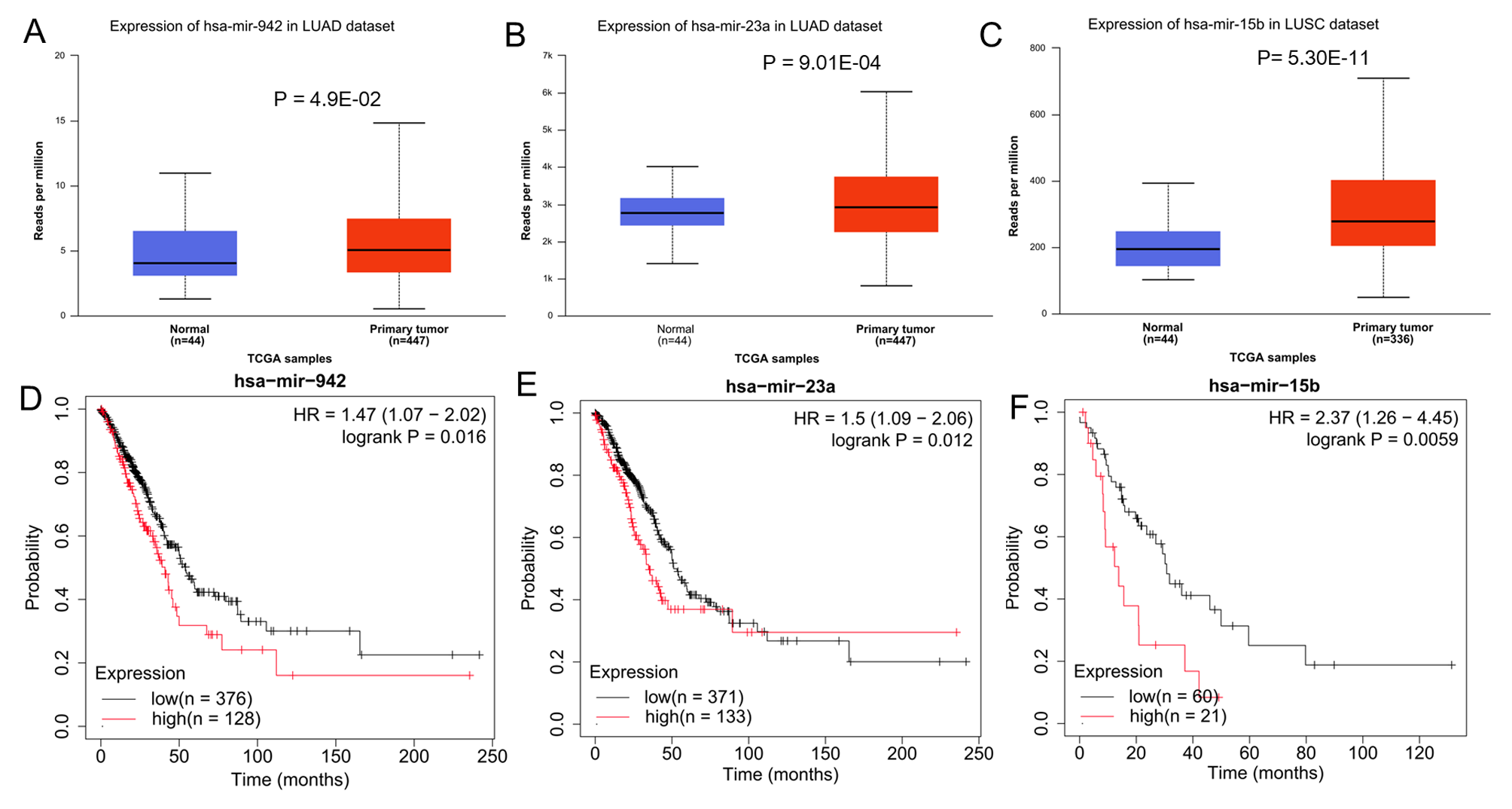

Supplement: Supplementary file 1 [file ijerph-19-02936-s001.zip › Figure S6.tif]

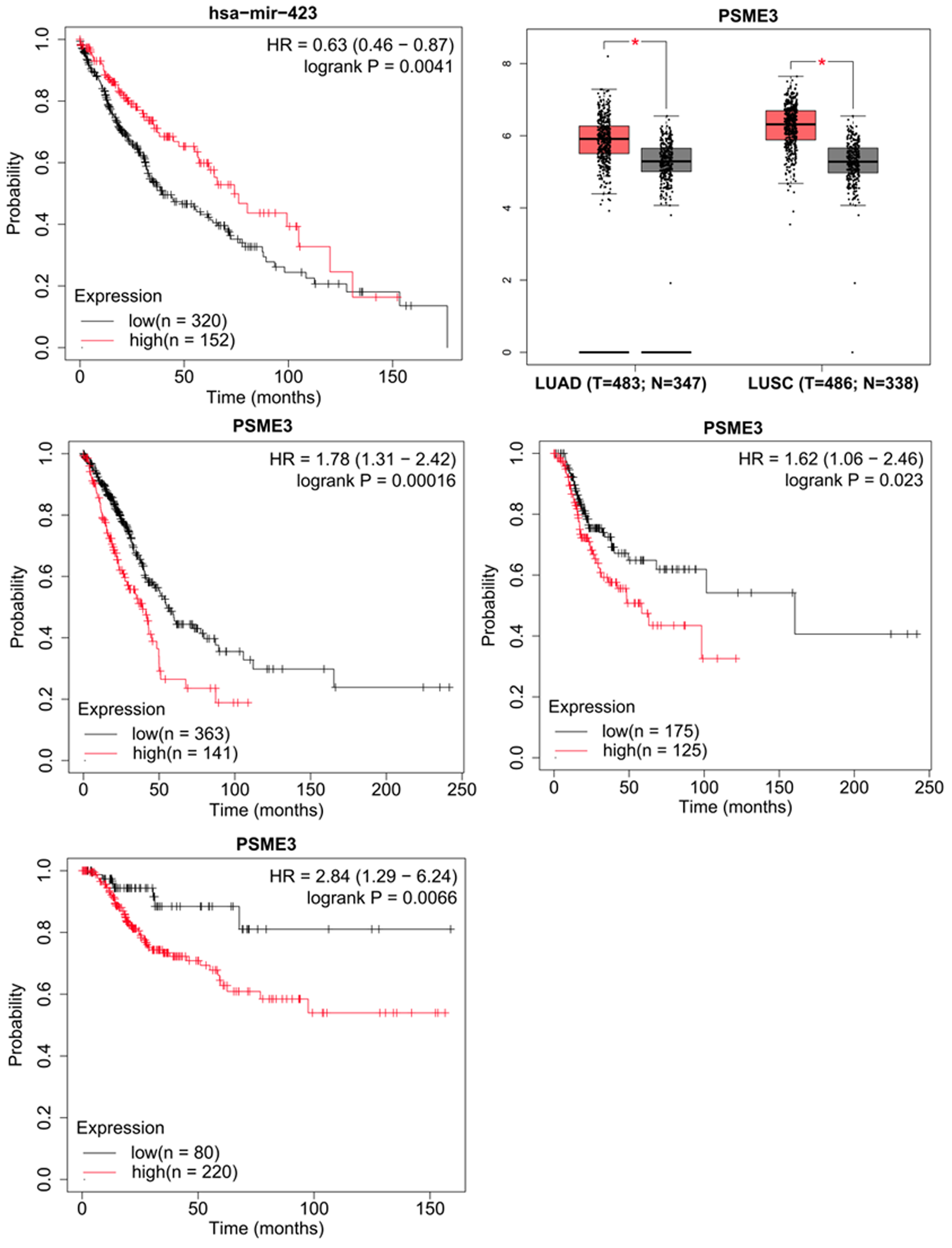

Supplement: Supplementary file 1 [file ijerph-19-02936-s001.zip › Figure S7.tif]
